# The Effect of Novel Oleanolic Acid Oximes Conjugated with Indomethacin on the Nrf2-ARE And NF-κB Signaling Pathways in Normal Hepatocytes and Human Hepatocellular Cancer Cells

**DOI:** 10.3390/ph14010032

**Published:** 2020-12-31

**Authors:** Maria Narożna, Violetta Krajka-Kuźniak, Barbara Bednarczyk-Cwynar, Robert Kleszcz, Wanda Baer-Dubowska

**Affiliations:** 1 Department of Pharmaceutical Biochemistry, Poznan University of Medical Sciences, 4, Święcicki Street, 60-781 Poznań, Poland; maria.narozna@ump.edu.pl (M.N.); vkrajka@ump.edu.pl (V.K.-K.); kleszcz@ump.edu.pl (R.K.); 2 Department of Organic Chemistry, Poznan University of Medical Sciences, 6, Grunwaldzka Street, 60-780 Poznań, Poland; bcwynar@ump.edu.pl

**Keywords:** oleanolic acid oxime derivatives, indomethacin conjugates, NF-κB, Nrf2, HepG2 cells, THLE-2 cells

## Abstract

Nrf2 and NF-κB play a key role in inflammation-driven cancers. Conjugation of anti-inflammatory drugs with oleanolic acid oxime (OAO) may enhance their therapeutic potential as a result of downregulation of these pathways. Novel OAO derivatives conjugated with indomethacin (IND) were synthesized, and their effect on the activation and expression of Nrf2 and NF-κB in HepG2 hepatoma cells and THLE-2 immortalized normal hepatocytes was evaluated in relation to cell cycle arrest and apoptosis. Treatment with OAO–IND conjugates reduced the activation of Nrf2 and NF-κB and the expression of their active forms in HepG2 cells, while in normal hepatocytes, the activation of Nrf2 was increased and NF-κB diminished. Compounds **3d**, 3-indomethacinoxyiminoolean-12-en-28-oic acid morpholide, and **3c**, 3-indomethacinoxyiminoolean-12-en-28-oic acid benzyl ester, were the most efficient. In THLE-2 cells, as opposed to HepG2 cells, the expressions of SOD-1 and NQO1 were significantly enhanced after treatment with these compounds. The COX-2 expression was diminished in both cell lines. OAO–IND derivatives affected the cell cycle arrest at G2/M, leading to increased apoptosis and increased number of resting HepG2 cells. Therefore, the conjugation of IND with OAO derivatives may preserve cancer cells against chemoresistance through the inhibition of the Nrf2-ARE pathway and NF-κB and, at the same time, exert a chemopreventive effect in normal hepatocytes.

## 1. Introduction

Naturally occurring triterpenoids, including oleanolic acid (OA), 3-β-hydroxyolean-12-en-28-oic acid, possess, besides other biological activities, an anti-inflammatory potential [1]. Prolonged inflammation resulting from viral hepatitis and/or metabolic disorders [2] is estimated to be the major cause of hepatocellular carcinoma (HCC) growth. Several studies have demonstrated that treatment with nonsteroidal anti-inflammatory drugs (NSAIDs) can reduce the incidence and mortality of a wide range of tumors, including HCC [3,4]. However, treatment with these drugs is often associated with adverse effects that mainly affect the gastrointestinal mucosa and cause liver dysfunction [5]. On the cellular level, oxidative stress has been implicated as a general mechanism in the toxicity of many NSAIDs [6]. It was reported that NSAIDs produce reactive oxygen species (ROS) inside cells [7], resulting in oxidative stress [8,9]. 

Several naturally occurring triterpenoids have been synthetically modified in order to increase their bioactivity and protective or therapeutic effects. Moreover, efforts to conjugate synthetic triterpenoids with NSAIDs have been performed. Such an approach may enhance their anti-inflammatory activity but also attenuate unfavorable NSAID side effects. 

The transcription factor NF-κB plays a key role in the inflammation process, controlling the expression of such genes as *COX-2* and *iNOS*. Overexpression of *COX-2*, which is related to risen cell growth and invasiveness, is discerned in human HCC. The Nrf2 signaling pathway, the major cellular defense against ROS and electrophilic species, negatively interferes with NF-κB signaling [10].

Our previous studies showed that new derivatives of oleanolic acid oxime (OAO) and particularly their conjugates with aspirin (ASP) downregulated COX-2 expression in HepG2 cells by modulating the NF-κB signaling pathway and suggested their potential application in the prevention of liver inflammation and cancer [11]. OAO derivatives also heightened the activation and expression of Nrf2 in HepG2 cells. This activity was correlated with their cytotoxicity. Still, conjugation with ASP enhanced Nrf2 activation in normal THLE-2 cells, while in HepG2 cells, this effect was less pronounced compared with that in nonconjugated OAO. Overall, these results suggested that OAO themselves, particularly OAO substituted with morpholide, may be deemed therapeutic agents, while OAO derivatives conjugated with ASP can be applied to liver cancer chemoprevention, supporting conventional treatment. Indomethacin is another widely used clinically NSAID. While aspirin represents salicylates, IND is methylated indole and belongs to the acetic acid derivative class of NSAIDs. Although both drugs are nonselective inhibitors of COX, early studies showed that aspirin is essentially an irreversible inhibitor of this enzyme, whereas the inhibition produced by IND is reversible [12]. Further studies reinforced this observation, showing that IND is a slow tight-binding inhibitor that establishes a rapidly reversible equilibrium with the enzyme, followed by a slow transition to a much more tightly bound COX–indomethacin complex. The formation of the tightly bound complex is responsible for indomethacin’s strong COX inhibitory activity [13]. Taking into consideration the differences in the mechanism of COX inhibition by these two NSAIDs, the aim of this study was to appraise the effect of the new synthesized conjugates of previously investigated OAO derivatives with indomethacin on Nrf2 and NF-κB signaling in connection with cell cycle distribution, apoptosis, and proliferation in normal hepatocytes and HCC cell lines.

## 2. Results and Discussion

### 2.1. Chemistry

**Scheme 1:**
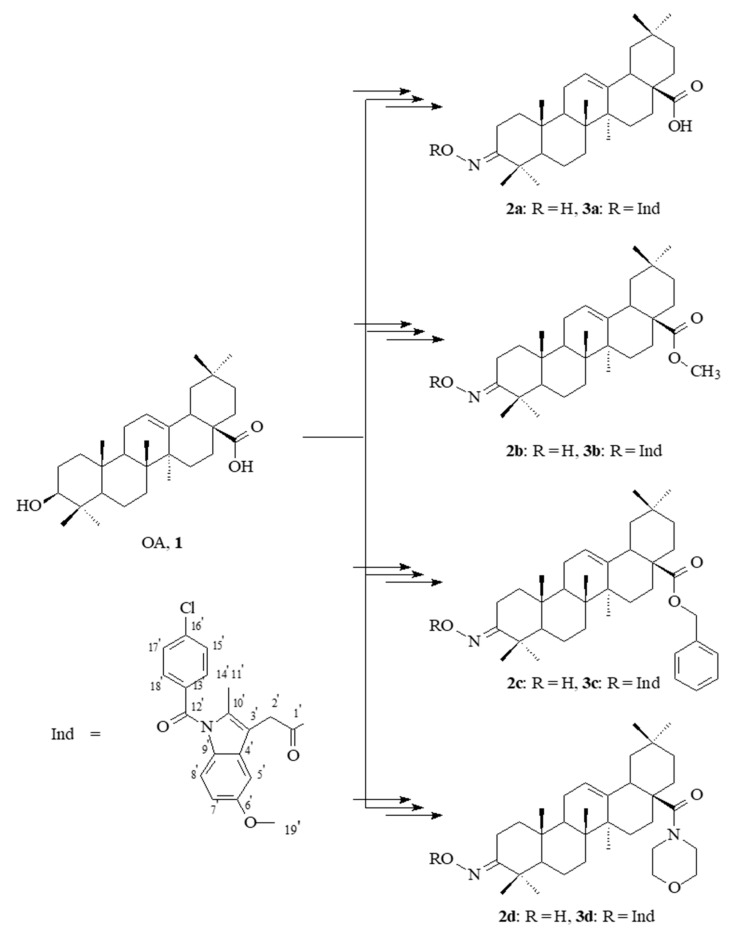
Synthesis of oleanolic acid oximes (**2a**–**d**) and their conjugates with IND (**3a**–**d**).

Oleanolic acid oxime derivatives and their conjugates were obtained using the earlier published methods [11,14,15,16,17]. Scheme 1 presents an overview of the synthesis.

### 2.2. Spectral Characteristics of the Oleanolic Acid Oximes and Their Conjugates with Indomethacin

The chemical structures of the synthesized compounds were elucidated based on IR, ^1^H NMR, ^13^C NMR, MS/EI, and elemental analysis.

In the IR spectra of conjugates **3a**–**d,** the presence of aromatic rings within the IND moiety was confirmed by the presence of an absorption band located at 3330–3335 cm^−1^. The signal observed at ν 1715 cm^−1^ was assigned to the C=O group within the –COO function of the indomethacin moiety. The next characteristic absorption band, observed at ν 1710 cm^−1^, was assigned to –N=C_-3_ of the acyloxyimino function.

The NMR analysis of conjugates **3a**–**d** confirmed the structures of these compounds. In the ^13^C NMR spectra of the above conjugates, the signals of an aromatic ring within the IND moiety (Cl–**C_6_H_4_**–CO–Ind–CH_2_–COON=C-_3_) were present at about δ 134 and 139 ppm (2 × C_q_, signal intensity: 1C), 132 ppm and 129 ppm (2 × CH, signal intensity: 2C). The C-12′ carbonyl carbon gave a signal that was observed at about 168 ppm. The aromatic IND carbon atoms (Cl–C_6_H_4_–CO–**Ind**–CH_2_–COON=C-_3_) formed signals, all of the intensity of 1C, located at the following values of chemical shifts: about δ 156, 136, and 131 ppm (3 × C_q_), 113, 112, and 101 ppm (3 × CH). The two last carbons of the indole system formed signals present at about δ 135 and 115 ppm (2 × C_q_). The carbon of the –CH_2_– group (between the indole system and acyl carbon, Cl–C_6_H_4_–CO–Ind–**CH_2_**–COON=C-_3_) was proved based on the presence of signal observed at about δ 30 ppm. The acyl carbon (Cl–C_6_H_4_–CO–Ind–CH_2_–**C**OON=C-_3_) gave the signal present at about δ 176 ppm. The two methyl group carbons (–O–C-_19′_–H_3_ and –C-_11′_–H_3_) gave signals observed at 55.7 and 13.3 ppm, respectively.

In the ^1^H NMR spectra of conjugates **3a**–**d**, the chlorinated aromatic protons (Cl–**C_6_H_4_**–CO–Ind–CH_2_–COON=C-**_3_**) formed two quartets, both intensity 2H, observed at δ 7.67 and 7.45 ppm. Three protons of the aromatic ring of the indole system (Cl–C_6_H_4_–CO–**Ind**–CH_2_–COON=C-_3_) formed two doublets (δ 6.97 and usually 6.89 ppm) and one doublet of doublets, present at δ 6.67–6.69 ppm. The protons of the –CH_2_– group (between indole system and acyl carbon, Cl–C_6_H_4_–CO–Ind–**CH_2_**–COON=C-_3_) formed a singlet of intensity 2H, observed at δ 3.60–3.61 ppm. For derivative **3d** (with the morpholide system), this signal was not observed, but a multiplet present in the range of 3.69–3.53 had intensity 10H: 2H from the –CH_2_– group and 8H from the morpholine ring. The two methyl group protons (–O–C-_19′_–H_3_ and –C-_11′_–H_3_) gave two singlets, observed at about δ 3.8 and 2.4 ppm, respectively.

All signals confirming the presence of oleanolate skeleton and characteristic functional groups were present in the ^1^H and ^13^C NMR spectra of conjugates **3a**–**d (**with the indomethacin moiety).

In the MS spectra of the conjugates **3a**–**d**, the presence of signals derived from mo-lecular ion (M^•+^) was observed. These signals were of low intensity (about 2–8%), which is quite often observed for triterpene derivatives of high molecular mass. Since the peak of the molecular ion corresponds to the molecular mass of the analyzed com-pound, the presence of such ion confirms the structure of the compound.

### 2.3. Effect of Indomethacin and Its Conjugates with OAO Derivatives on Cell Viability

**Figure 1:**
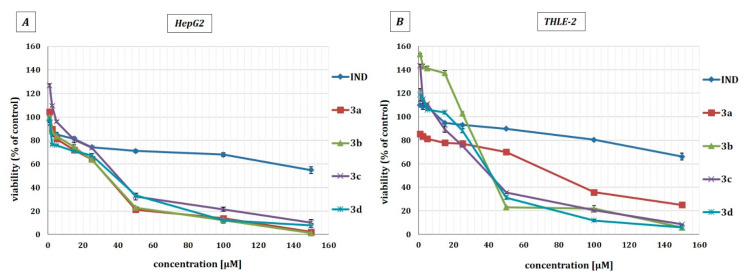
The effect of IND and OAO–IND derivatives (3a–d) on the viability of HepG2 panel (A) and THLE-2 panel (B) cells. Data (means ± SEM) from three separate experiments are presented.

The impact of IND and OAO–IND derivatives (**3a**–**d**) on the viability of HepG2 hepatoma cells and human immortalized hepatocytes (THLE-2) was evaluated using the MTT assay. As shown in Figure 1 in the concentration range of 1–150 µM, all tested compounds reduced both cell types’ viability in a dose-dependent manner. All conjugates demonstrated higher cytotoxicity than the IND, but within the tested concentration range of 60–70%, cell viability was assured. Compound **3a** showed lower cytotoxicity to THLE-2 cells than to HepG2 cells and in comparison with the other conjugates. The analysis of IC_50_ values indicated that OAO–IND derivatives are slightly more cytotoxic to hepatoma HepG2 cells than to immortalized normal hepatocytes (Table 1). Overall, these data indicate that OAO–IND derivatives may be more efficient as therapeutic agents than the indomethacin itself.

### 2.4. The Effect of Indomethacin and Its Conjugates with OAO Derivatives on Nrf2 Activation and Expression of Its Target Genes

Based on the results of the MTT test in further assays, concentrations of 10 and 20 µM of IND and its conjugates with OAO derivatives were applied. The IND concentrations used in this study were similar to those applied by other authors in in vitro studies and were relevant to clinical doses. 

The activation of Nrf2 was evaluated based on the amount of Nrf2 contained in the DNA-binding complex to the ARE sequence. The oligonucleotides containing the ARE consensus-binding site (5′-GTCACAGTGACTCAGCAGAATCTG-3′) for Nrf2 were immobilized on microplates as bait.

**Figure 2:**
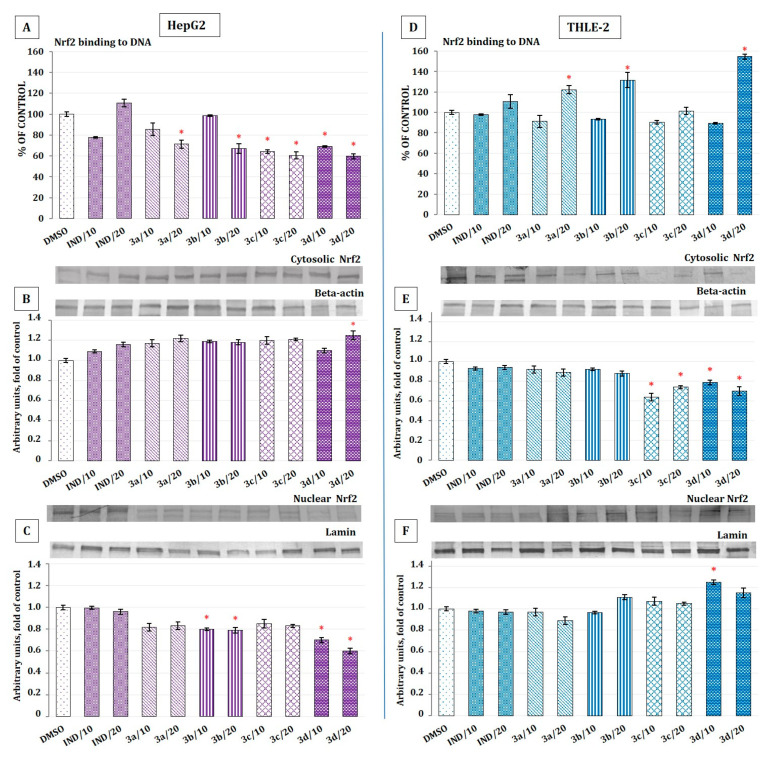
The effect of IND and OAO–IND derivatives (3a–d) on the Nrf2–DNA binding capability and translocation from cytosol to nuclei in HepG2 panels (A–C) and THLE-2 panels (D–F) cells. Activated Nrf2 was assessed in terms of the amount of Nrf2 contained in the DNA-binding complexes extracted from the nuclear fraction panels (**A**,**D**). Data (means ± SEM) from three separate experiments are presented (expression equals 100%). Representative immunoblots for the analysis of the cytosolic panels (**B**,**E**) and nuclear panels (**C**,**F**) levels of Nrf2 protein are shown. The sequence of the bands corresponds to the sequence of the bars in the graph. Lamin and β-actin content were used for the normalization of the results. The values were calculated as protein level in comparison with control cells (expression equals 1). * Significantly different from DMSO (control)-treated cells, *p* < 0.05.

As shown in Figure 2A, in HepG2 cells all conjugates decreased the Nrf2 binding level, while IND tended to increase it. The most pronounced effect, a decrease in binding level by ~40%, was found in the case of the OAO–IND morpholide derivative **3d**.

In THLE-2 cells, a significant increase in Nrf2 binding level was observed as a result of treatment with carboxylic (**3a**), methyl ester (**3b**), and morpholide (**3d**) derivatives by ~22%, ~32%, and ~55%, respectively, at higher concentrations of 20 µM (Figure 2D). 

Nrf2 under normal conditions is bound in the cytosol by the Keap1 protein, which recruits the ubiquitin ligase complex and targets Nrf2 for proteasomal degradation. Stress conditions or the other stimuli, including well-known chemopreventive agents, induce the modification of key cysteine residues in Keap1, resulting in a conformational change that prevents Nrf2 ubiquitination and allows its translocation to the nucleus, and binding to the target ARE sequence [18].

The reduced activation of Nrf2 in HepG2 cells and increased activation in THLE-2 cells as a result of treatment with OAO–IND conjugates was further confirmed by Nrf2 translocation from cytosol to nucleus (Figure 2B,C,E,F). Again, conjugate **3d** was shown to be the most effective modulator of Nrf2 translocation.

**Figure 3:**
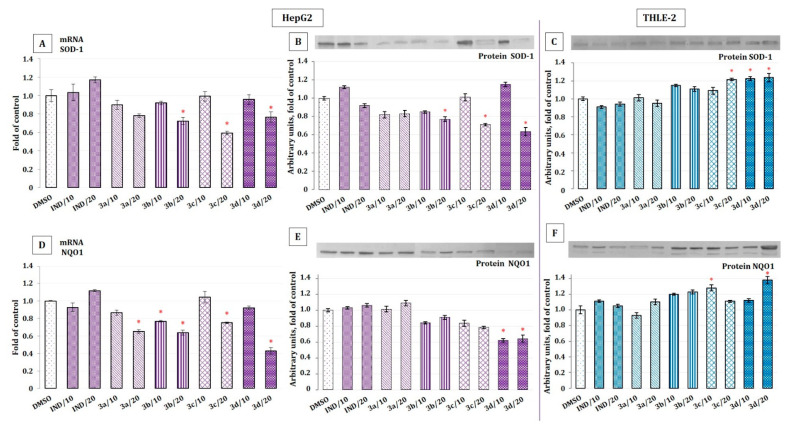
The effect of IND and OAO–IND derivatives (3a–d) on SOD-1 panels (A–C) and NQO1 panels (D–F) mRNA and protein level in HepG2 and THLE-2 cells. The values (means ± SEM) for the mRNA panels (**A**,**D**) levels were calculated from three separate experiments in comparison with control cells (expression equals 1). Representative immunoblots of the cytosolic content of SOD-1 and NQO1 in HepG2 panels (**B**,**E**) and THLE-2 cells panels (**C**,**F**) from three separate experiments are shown. The sequence of the bands corresponds to the sequence of the bars in the graph. The values (means ± SEM) were calculated as protein level in comparison with control cells (expression equals 1). * Significantly different from DMSO (control)-treated cells, *p* < 0.05.

Nrf2 controls the cellular signaling involved in the transcriptional activation of genes encoding a panel of antioxidant/phase 2 detoxifying enzymes and stress-responsive proteins [18]. Therefore, Nrf2 plays an essential role in maintaining cellular homeostasis and hence represents a critical target for the chemoprevention of oxidative stress- or inflammation-associated carcinogenesis. In this study, as a result of Nrf2 activation by conjugate **3d**, increased protein levels of SOD-1 and NQO1 genes, encoding antioxidant and phase 2 enzyme, respectively, in immortalized hepatic normal cells were observed (Figure 3C,F), indicating its possible application in liver cancer chemoprevention. 

In contrast, in HepG2 cells, the same compound along with **3b** and **3c** reduced the protein levels of SOD-1 and NQO1 along with transcripts of genes encoding these enzymes (Figure 3A,B,D,E). Again, compound **3d** was the most effective. In HepG2 cells derived from human hepatocellular carcinoma, this effect is highly desirable since the constitutive activation of Nrf2 in cancer cells has been implicated in cancer progression and in resistance to cancer chemo- and radiotherapy. 

IND basically did not show any effect on the Nrf2 activation, neither in THLE-2 nor in HepG2 cells.

This observation suggests that conjugation with derivatives of OAO may improve its pharmacological activities in both chemoprevention and chemotherapeutic contexts.

### 2.5. The Effect of Indomethacin and Its Conjugates with OAO Derivatives on NF-κB Activation and Expression of COX-2 Gene 

Pharmacological and genetic studies suggest functional cross-talk between Nrf2 and NF-κB, the two key transcription factors that regulate cellular responses to oxidative stress and inflammation, respectively. The absence of Nrf2 can exacerbate NF-κB activity, leading to increased cytokine production, whereas NF-κB can modulate Nrf2 transcription and activity, having both positive and negative effects on the target gene expression.

Therefore, in this study, the effect of OAO–IND conjugates on the activation of NF-κB was also assessed to find a possible relationship between the impacts of these compounds on both signaling pathways. The NF-κB family is composed of five proteins—RelA (p65), RelB, c-Rel, NF-B1 (p50), and NF-B2 (p52)—each of which may form homo- or heterodimers. The term NF-κB commonly refers to a p50–p65 heterodimer, which represents the major Rel/NF-κB complex in most cells [19]. In unstimulated cells, NF-κB is sequestered in the cytoplasm through a tight association with its inhibitor, IκB protein. The NF-κB activation most commonly occurs through the site-specific phosphorylation of IκB by IκB kinase beta (IKK), leading to a degradation of the inhibitory subunit by the 26S proteasome, which allows free NF-κB heterodimers to migrate to the cell nucleus and to bind specific consensus sequences in the target genes [20]. 

**Figure 4:**
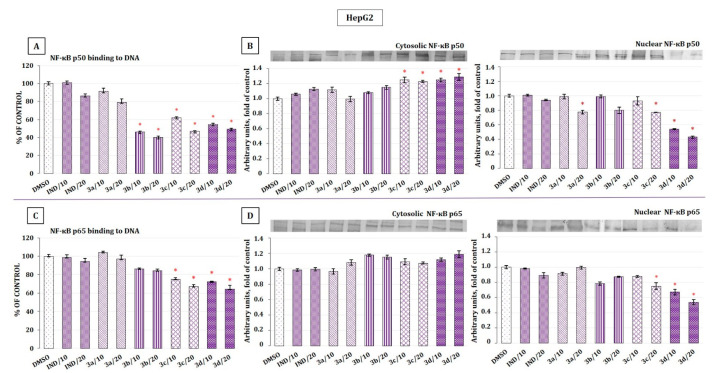
The effect of IND and OAO–IND derivatives (3a–d) on the NF-κB p50 and NF-κB p65 binding to DNA panels (**A**,**C**) and translocation from cytosol to nuclei in HepG2 cells panels (**B**,**D**). Activated NF-κB p50 and NF-κB p65 were assessed in terms of the amount of NF-κB p50 panel (**A**) and NF-κB p65 panel (**C**) contained in the DNA-binding complexes extracted from the nuclear fraction. The values (means ± SEM) were calculated from three separate experiments as protein level in comparison with control cells (expression equals 100%). Representative immunoblots for the analysis of the cytosolic and nuclear levels of proteins NF-κB p50 panel (**B**) and NF-κB p65 panel (**D**) are shown. The sequence of the bands corresponds to the sequence of the bars in the graph. The values were calculated as protein level in comparison with control cells (expression equals 1). * Significantly different from DMSO (control)-treated cells, *p* < 0.05.

Figure 4 presents the results of the assessment of the binding of p50 and p65 NF-κB subunits to their immobilized consensus site and translocation from cytosol to nucleus in HepG2 cells. All OAO–IND derivatives, with the exception of **3a** and in contrast to IND, diminished the content of NF-κB p50 and NF-κB p65 subunits in the DNA-binding complex extracted from the cell nuclei. The 40–60% and 25–35% reductions of the binding levels of p50 and p65, respectively, were observed as a result of treatment with methyl ester (3b), benzyl ester (3c), and morpholide (3d) derivatives (Figure 4A,C). The reduced activation of NF-κB as a result of treatment with **3c** and **3d** was further confirmed by diminished levels of p50 and p65 nuclear proteins.

**Figure 5:**
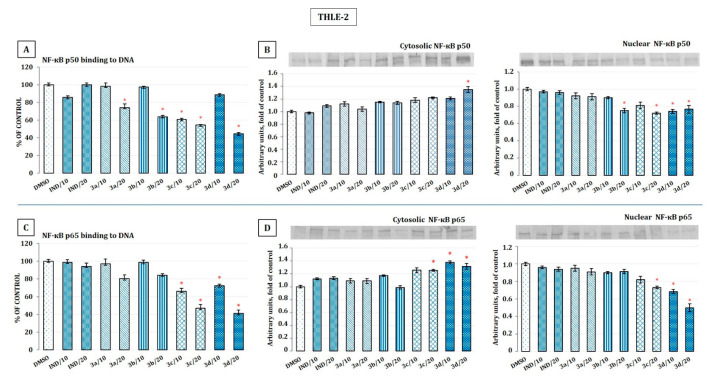
The effect of IND and OAO–IND derivatives (3a–d) on the NF-κB p50 and NF-κB p65 binding to DNA panels (**A**,**C**) and translocation from cytosol to nuclei in THLE-2 cells panels (**B**,**D**). Activated NF-κB p50 and NF-κB p65 were assessed in terms of the amount of NF-κB p50 panel (**A**) and NF-κB p65 panel (**C**) contained in the DNA-binding complexes extracted from the nuclear fraction. The values (means ± SEM) were calculated from three separate experiments as protein level in comparison with control cells (expression equals 100%). Representative immunoblots for the analysis of the cytosolic and nuclear levels of proteins NF-κB p50 panel (**B**) and NF-κB p65 panel (**D**) are shown. The sequence of the bands corresponds to the sequence of the bars in the graph. The values were calculated from three separate experiments as protein level in comparison with control cells (expression equals 1). * Significantly different from DMSO (control)-treated cells, *p* < 0.05.

The same conjugates of IND (i.e., benzyl ester (**3c**) and morpholide (**3d**) derivatives) were also the most effective NF-κB activation inhibitors in THLE-2 cells. As shown in Figure 5A,C, reduced binding levels by about 25–55% and 28–60% for p50 and p65, respectively, compared with the control group were found as a result of treatment with these compounds. The changes in the binding patterns of p50 and p65 subunits to their consensus side were related to their reduced translocation, particularly diminished nuclear protein levels. Here, evidently, compound 3d affected more efficiently the translocation of p65 than that of p50 (Figure 5B,D).

The NF-κB p50 subunit serves as a helper in NF-κB DNA binding and acts predominantly as a control subunit of the NF-κB complex, whereas the p65 subunit is responsible for initiating the transcription [21]. Hence, NF-κB p65 was so far considered a major point for drug discovery and development [22]. However, more recent studies indicated that p50 could potentially regulate such important cellular events as apoptosis independently of the NF-κB complex. Therefore, p50 can also be a target for cancer prevention and therapy [23].

**Figure 6:**
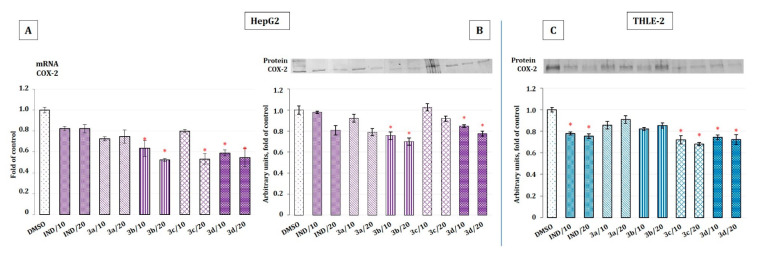
The effect of IND and OAO–IND derivatives (3a–d) on the level of *COX-2* mRNA panel (A) and protein level in HepG2 panel (B) and THLE-2 panel (C) cells. The values (means ± SEM) for the mRNA level of COX-2 in HepG2 cells panel (**A**) were calculated from three separate experiments in comparison with control cells (expression equals 1). Representative immunoblots of the cytosolic content of COX-2 in HepG2 panel (**B**) and THLE-2 cells panel (**C**) from three separate experiments are shown. The sequence of the bands corresponds to the sequence of the bars in the graph. The values (means ± SEM) were calculated as protein level in comparison with control cells (expression equals 1). * Significantly different from DMSO (control)-treated cells, *p* < 0.05.

*COX-2* is one of the major genes under the NF-κB transcriptional control. Moreover, overexpression of *COX-2* was found in human HCC [11]. Consequently, reduced NF-κB activation, particularly as a result of treatment with compound **3d** in both cell lines’ diminished level of the COX-2 protein, was observed (Figure 6). In HepG2 cells, a slightly more pronounced effect was noticed in the case of conjugate **3b** and was confirmed on the transcription level. In these cells, the impact of IND was statistically insignificant, while in THLE-2 cells, the difference in COX-2 levels in IND treated in comparison with control cells achieved statistical significance. In THLE-2 cells, in contrast to HepG2 cells, compound **3c** was more efficient in reducing COX-2 expression than the other conjugates.

Overall, these data indicate that OAO–IND conjugates, particularly benzyl ester and morpholide derivatives of OAO–IND, are more efficient inhibitors of NF-κB in both normal immortalized hepatocytes and HCC HepG2 cells than IND. The latter at the same concentration showed a much weaker effect. Therefore, the conjugation of IND with these compounds may improve its anti-inflammatory activity.

### 2.6. The Effect of Indomethacin and Its Conjugates with OAO Derivatives on the Nrf2 and NF-κB Subunits’ Transcript Levels in HepG2 Cells

To find out whether the tested IND conjugates affect Nrf2 and NF-κB expressions, their transcripts were evaluated in HepG2 cells. All conjugates at a concentration of 20 µM reduced Nrf2 mRNA levels, while IND tended to increase them, but the difference compared with control was not statistically significant. Compound **3d** diminished the transcript level of both NF-κB subunits, but to a greater extent, p50, which was also affected as a result of treatment with conjugate **3c**. 

**Figure 7:**
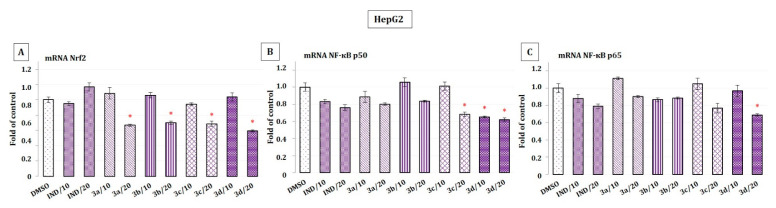
The effects of IND and OAO–IND derivatives (**3a**–**d**) on the Nrf2 panel (**A**) and NF-κB subunits’ panels (**B**,**C**) transcript levels in HepG2 cells. The values (means ± SEM) for the mRNA in HepG2 cells were calculated from three separate experiments in comparison with control cells (expression equals 1). * Significantly different from DMSO (control)-treated cells, *p* < 0.05.

IND did not change significantly the transcript level of any NF-κB subunits (Figure 7).

These results show that OAO–IND conjugates, particularly compound **3d** in HepG2 cells, not only diminish the activation of the key signaling pathways involved in HCC development but also affect the expression of the transcription factors participating in the final stages of these pathways. An evident link was shown between the downregulations of both pathways by OAO–IND conjugates. The explanation of the mechanism of this interrelation requires further studies.

### 2.7. The Effect of Indomethacin and Its Conjugates with OAO on Cell Cycle Distribution, Apoptosis, and Proliferation of HepG2 Cells

Evidence shows that NF-κB activation can enhance specific anti-apoptotic gene expression, which then induces cell survival. On the contrary, the downregulation of NF-κB activates pro-apoptotic genes [24]. Moreover, analyses of the genome-wide distribution of Nrf2 have identified sets of Nrf2 target genes whose products are involved in cell proliferation and differentiation [25]. Therefore, the downregulations of both pathways observed in this study by OAO–IND suggest their possible anti-apoptotic and/or antiproliferative effect.

**Figure 8:**
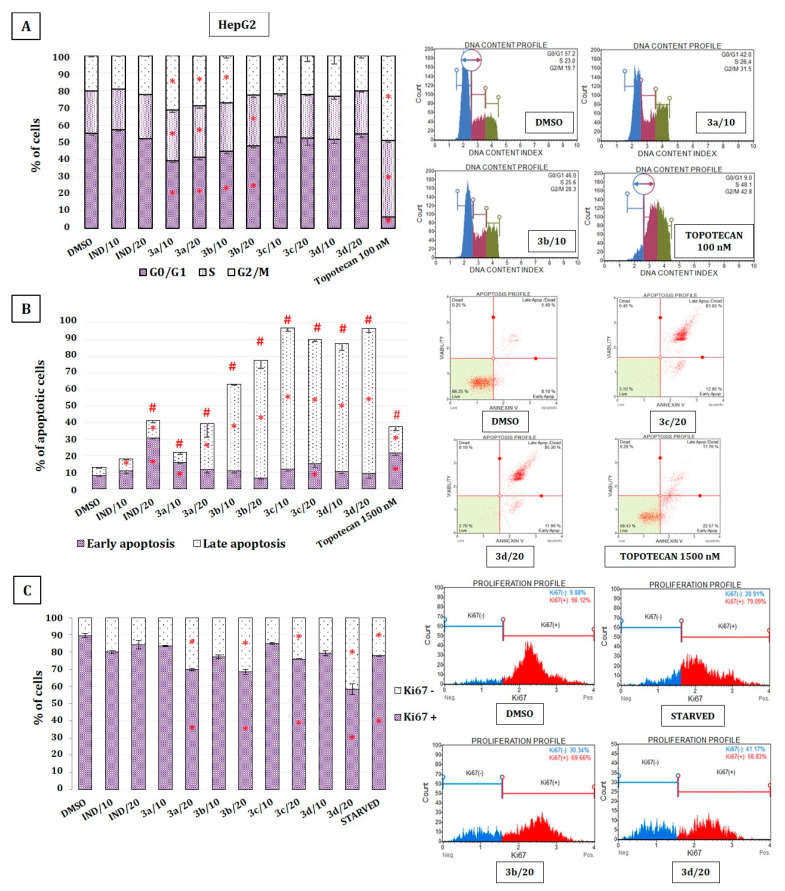
The effect of IND and OAO–IND derivatives (**3a**–**d**) on the cell cycle distribution panel (**A**), apoptosis induction panel (**B**), and proliferation level panel (**C**) in HepG2 cells. Panel (**A**): Graphs represent the percentage of cells in the G1/G0, S, and G2/M phases measured by the flow cytometry after propidium iodide staining. Topotecan was used as a positive control. Representative plots are presented. Panel (**B**): Graphs represent the percentage of cells in the early and late stages of the apoptosis assessed by the flow cytometry measurements based on the signal from annexin V bound to phosphatidylserine externalized in apoptotic cells. A dead cell marker, 7-AAD, was also included. Topotecan was used as a reference for pro-apoptotic activity. Representative plots are presented. Panel (**C**): graphs represent the percentage of proliferating (Ki67(+)) and nonproliferating (Ki67(−)) cells measured by the flow cytometry. Starved cells (cultured in FBS-free medium) were used as a reference to antiproliferative conditions. Representative plots are presented. For all panels, mean values ± SEM from three independent experiments are shown. Asterisks (*) denote statistically significant changes from DMSO (control)-treated cells, and for panel B denote statistically significant changes in the percentage of either early or late apoptotic cells, while hashes (#) denote statistically significant changes in the percentage of total apoptotic cells, *p* < 0.05.

As shown in Figure 8A, OAO–IND carboxylic derivative 3a and compound **3b** increased the amounts of cells in G2/M and decreased in G0/G1 phases. However, compared with topotecan, a reference compound, which in low concentration induces cell cycle arrest in S and G2/M phases, the effect of OAO–IND conjugates was much weaker. 

All of the evaluated compounds increased the number of apoptotic cells, and this effect was, in most cases, even more pronounced than that observed after treatment with topotecan (Figure 8B). The highest percentage of total apoptotic cells was found as an effect of treatment with OAO–IND derivatives **3b**, **3c**, and **3d** (by ~64%, ~84%, and ~83%, respectively, in comparison with the results from DMSO-treated cells). In general, IND was a much less effective apoptosis inducer, but in contrast to its conjugates, it increased mostly the percentage of the cells in the early stage of apoptosis. The effect of OAO–IND conjugates on cell proliferation was evaluated based on the Ki67 protein expression, allowing the assessment of the cells’ proliferation rate. The most pronounced antiproliferative effect among the tested derivatives was observed as a result of treatment with compounds **3b** and **3d**, differing in methyl ester and morpholide groups at the C-17 position of OAO molecules. In this regard, the highest percentage of resting cells (cells exhibiting the lowest Ki67 expression) was found after treatment with OAO morpholide **3d** and OAO methyl ester **3b**. The percentage of resting cells treated with these compounds exceeded the amount observed in starved cells lacking FBS in the culture medium (Figure 8C).

## 3. Materials and Methods

### 3.1. Chemistry

General information concerning the performed chemical experiments as well as concerning the elucidation of the synthesized compounds’ chemical structures is presented in our earlier publication [11]. The purity of the obtained compounds was evaluated based on spectral data (NMR and elemental analysis). Copies of ^1^H NMR and ^13^C NMR spectra for all final compounds are attached in the Supplementary Materials (Figures S1–S8).

Conjugate of oleanolic acid oxime and indomethacin, 3-indomethacinoxyiminoolean-12-en-28-oic acid (**3a**):

**C_49_H_61_ClN_2_O_6_**. **Mol. mass**: 808.422. **Yield**: 745 mg (92.2 %). M.p.: 115–119 °C, crystalline powder. **IR** (KBr, cm^−1^) ν: 3330 (CH, Cl–**C_6_H_4_**–CO–**Ind**–CH_2_–COON=C-_3_), 1715 (C=O, Cl–C_6_H_4_–CO–Ind–CH_2_–**CO**ON=C-_3_); 1710 (N=C, Cl–C_6_H_4_–CO–Ind–CH_2_–COO**N=C**-_3_); 1700 (C=O, **CO**OH); Ind = indole system. **^13^C NMR** (151 MHz, CDCl_3_) δ: 183.66 (C_q_, –COOH, C-28); 175.44 (C_q_, Cl–C_6_H_4_–CO–Ind–CH_2_–**CO**ON=C-_3_, C-1′); 168.31 (C_q_, Cl–C_6_H_4_–**CO**–Ind–CH_2_–COON=C-_3_, C-12′); 162.47 (C_q_, C-3); 156.06 (C_q_, Cl–C_6_H_4_–CO–**Ind**–CH_2_–COON=C-_3_, C-6′); 143.83 (C_q_, C–13); 139.38 (C_q_, Cl–**C_6_H_4_**–CO–Ind–CH_2_–COON=C-_3_, C-16′); 136.29 (C_q_, Cl–C_6_H_4_–CO–**Ind**–CH_2_–COON=C-_3_, C-4′); 135.22 (C_q_, Cl–C_6_H_4_–CO–**Ind**–CH_2_–COON=C-_3_, C-10′); 133.86 (C_q_, Cl–**C_6_H_4_**–CO–Ind–CH_2_–COON=C-_3_, C-13′); 132.08 × 2 (2 × CH, Cl–**C_6_H_4_**–CO–Ind–CH_2_–COON=C-_3_, C-14′ and C-18′); 130.83 (C_q_, Cl–C_6_H_4_–CO–**Ind**–CH_2_–COON=C-_3_, C-9′); 129.08 × 2 (2 × CH, Cl–**C_6_H_4_**–CO–Ind–CH_2_–COON=C-_3_, C-15′ and C-17′); 122.35 (CH, C-12); 114.96 (C_q_, Cl–C_6_H_4_–CO–**Ind**–CH_2_–COON=C-_3_, C-3′); 113.49 (CH, Cl–C_6_H_4_–CO–**Ind**–CH_2_–COON=C-_3_, C-8′); 111.74 (CH, Cl–C_6_H_4_–CO–**Ind**–CH_2_–COON=C-_3_, C-7′); 101.15 (CH, Cl–C_6_H_4_–CO–**Ind**–CH_2_–COON=C-_3_, C-5′); 55.75 (CH_3_, –OCH_3_, C-19′); 46.66 (C_q_, C-17); 29.97 (CH_2_, Cl–C_6_H_4_–CO–Ind–**CH_2_**–COON=C-_3_, C-2′); 13.32 (CH_3_, C-11′); Ind = indole system. **^1^H NMR** (600 MHz, CDCl_3_) δ: 7.67 (2H, q_a_, *J* = 9.0 Hz, Cl–**C_6_H_4_**–CO–Ind–CH_2_–COON=C-_3_, C-_14′_–H and C-_18′_–H); 7.44 (2H, q_a_, *J* = 9.0 Hz, Cl–**C_6_H_4_**–CO–Ind–CH_2_–COON=C-_3_, C-_15′_-H and C-_17′_-H); 6.97 (1H, d, *J* = 2.4 Hz, Cl–C_6_H_4_–CO–**Ind**–CH_2_–COON=C-_3_, C-_8′_–H); 6.89 (1H, d, *J* = 9.0 Hz, Cl–C_6_H_4_–CO–**Ind**–CH_2_–COON=C-_3_, C-_5′_–H); 6.69 (1H, dd, *J* = 9.0, and 2.4 Hz, Cl–C_6_H_4_–CO–**Ind**–CH_2_–COON=C-_3_, C-_7′_–H); 5.28 (1H, t, *J* = 3.4 Hz, C_12_–H); 3.84 (3H, s, –OCH_3_, C-_19′_–H_3_); 3.61 (2H, s, Cl–C_6_H_4_–CO–Ind–**CH_2_**–COON=C-_3_, C-_2′_–H_2_); 2.86 (1H, dd, *J* = 13.8, and 4.1 Hz, C-_18_–H_β_); 2.38 (3H, s, CH_3_, C-_11′_–H_3_); 1.18, 1.04, 1.00, 0.96, 0.85, 0.93, 0.78 (7 × 3H, 7 × s, 7 × CH_3_ groups); Ind = indole system. **MS/EI** (*m*/*z*, %): 15.02 (12%, CH_3_), 112.01 (9%, C_6_H_4_Cl), 138.99 (100%, Cl–C_6_H_4_CO, main ion), 173.09 (6%, 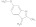), 357.79 (6%, indomethacin), 406.34 (39%, 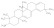), 790,41 (9%, 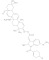), 792.40 (5%, 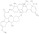), 808.42 (4%, M^•+^, molecular ion). **Elem. anal.**: for C_49_H_61_ClN_2_O_6_ calcd.: C: 72.71%, H: 7.60%, N: 3.46%, found: C: 72.73%, H: 7.59, %, N: 3.48%.

Conjugate of methyl oleanolate oxime and indomethacin, 3-indomethacinoxyiminoolean-12-en-28-oic acid methyl ester (**3b**): 

**C_50_H_63_ClN_2_O_6_**. **Mol. mass**: 822.437. **Yield**: 781 mg (95.0 %). **M.p.**: 80–85 °C, amorphous powder. **IR** (KBr, cm^−1^) ν: 3335 (CH, Cl–**C_6_H_4_**–CO–**Ind**–CH_2_–COON=C-_3_), 1725 (C=O, –**CO**OCH_3_), 1715 (C=O, Cl–C_6_H_4_–CO–Ind–CH_2_–**CO**ON=C-_3_); 1710 (N=C**,** Cl–C_6_H_4_–CO–Ind–CH_2_–COO**N=C**-_3_); Ind = indole system. **^13^C NMR** (151 MHz, CDCl_3_) δ: 178.26 (C_q_, –**CO**OCH_3_, C-28); 175.45 (C_q_, Cl–C_6_H_4_–CO–Ind–CH_2_–**CO**ON=C-_3_, C-1′); 168.30 (C_q_, Cl–C_6_H_4_–**CO**–Ind–CH_2_–COON=C-_3_, C-12′); 162.45 (C_q_, C-3); 156.04 (C_q_, Cl–C_6_H_4_–CO–**Ind**–CH_2_–COON=C-_3_, C-6′); 143.93 (C_q_, C-13); 139.44 (C_q_, Cl–**C_6_H_4_**–CO–Ind–CH_2_–COON=C-_3_, C-16′); 136.23 (C_q_, Cl–C_6_H_4_–CO–**Ind**–CH_2_–COON=C-_3_, C-4′); 135.24 (C_q_, Cl–C_6_H_4_–CO–**Ind**–CH_2_–COON=C-_3_, C-10′); 133.87 (C_q_, Cl–**C_6_H_4_**–CO–Ind–CH_2_–COON=C-_3_, C-13′); 132.15 × 2 (2 × CH, Cl–**C_6_H_4_**–CO–Ind–CH_2_–COON=C-_3_, C-14′ and C-18′); 130.92 (C_q_, Cl–C_6_H_4_–CO–**Ind**–CH_2_–COON=C-_3_, C-9′); 129.11 × 2 (2 × CH, Cl–**C_6_H_4_**–CO–Ind–CH_2_–COON=C-_3_, C-15′ and C-17′); 122.05 (CH, C-12); 115.00 (C_q_, Cl–C_6_H_4_–CO–**Ind**–CH_2_–COON=C-_3_, C-3′); 113.44 (CH, Cl–C_6_H_4_–CO–**Ind**–CH_2_–COON=C-_3_, C-8′); 111.80 (CH, Cl–C_6_H_4_–CO–**Ind**–CH_2_–COON=C-_3_, C-7′); 101.20 (CH, Cl–C_6_H_4_–CO–**Ind**–CH_2_–COON=C-_3_, C-5′); 55.75 (CH_3_, –OCH_3_, C-19′); 51.50 (CH_3_, –COO**CH_3_**); 45.81 (C_q_, C-17); 29.89 (CH_2_, Cl–C_6_H_4_–CO–Ind–**CH_2_**–COON=C-_3_, C-2′); 13.32 (CH_3_, C-11′); Ind = indole system. **^1^H NMR** (600 MHz, CDCl_3_) δ: 7.67 (2H, q_a_, *J* = 9.0 Hz, Cl–**C_6_H_4_**–CO–Ind–CH_2_–COON=C-_3_, C-_14′_–H and C-_18′_–H); 7.45 (2H, q_a_, *J* = 9.0 Hz, Cl–**C_6_H_4_**–CO–Ind–CH_2_–COON=C-_3_, C-_15′_–H and C-_17′_–H); 6.97 (1H, d, *J* = 2.4 Hz, Cl–C_6_H_4_–CO–**Ind**–CH_2_–COON=C-_3_, C-_8′_-H); 6.88 (1H, d, *J* = 9.0 Hz, Cl–C_6_H_4_–CO–**Ind**–CH_2_–COON=C-_3_, C-_5′_–H); 6.69 (1H, dd, *J* = 9.0, and 2.4 Hz, Cl–C_6_H_4_–CO–**Ind**–CH_2_–COON=C-_3_, C-_7′_-H); 5.31 (1H, t, *J* = 3.1 Hz, C_12_–H); 3.84 (3H, s, –OCH_3_, C-_19′_–H_3_); 3.65 (3H, s, –COO**CH_3_**); 3.61 (2H, s, Cl–C_6_H_4_–CO–Ind–**CH_2_**–COON=C-_3_, C-_2′_–H_2_); 2.85 (1H, dd, *J* = 13.8, and 4.0 Hz, C-_18_–H_β_); 2.38 (3H, s, CH_3_, C-_11′_–H_3_); 1.16, 1.03, 0.99, 0.95, 0.92, 0.91, 0.78 (7 × 3H, 7 × s, 7 × CH_3_ groups); Ind = indole system. **MS/EI** (*m*/*z*): 15.02 (12%, CH_3_), 60.03 (21%, COOCH_3_), 112.01 (9%, C_6_H_4_Cl), 138.99 (99%, Cl–C_6_H_4_CO, main ion), 173.09 (4%, 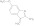), 357.79 (9%, indomethacin), 406.34 (34%, 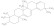), 790,41 (13%, 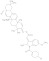), 746.43 (4%, 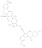), 822.51 (5%, M^•+^, molecular ion). **Elem. anal.**: for C_50_H_63_ClN_2_O_6_ calcd.: C: 72.93%, H: 7.71%, N: 3.40%, found: C: 72.90%, H: 7.70, %, N: 3.42%.

Conjugate of benzyl oleanolate oxime and indomethacin, *3-indomethacinoxyiminoolean-12-en-28-oic acid benzyl ester* (**3c**)**:**

**C_56_H_67_ClN_2_O_6_**. **Mol. mass**: 898.469. **Yield**: 846 mg (94.2 %). **M.p.**: 130–133 °C, needles (EtOH). **IR** (KBr, cm^−1^): 3330 (CH, Cl–**C_6_H_4_**–CO–**Ind**–CH_2_–COON=C-_3_), 2900 (CH, –COOCH_2_**C_6_H_5_**); 1715 (C=O, Cl–C_6_H_4_–CO–Ind–CH_2_–**CO**ON=C-_3_); 1710 (N=C, Cl–C_6_H_4_–CO–Ind–CH_2_–COO**N=C**-_3_); 1705 (C=O, –**CO**OCH_2_C_6_H_5_); Ind = indole system. **^13^C NMR** (151 MHz, CDCl_3_) δ: 177.36 (C_q_, –**CO**OCH_2_–C_6_H_5_, C-28); 175.48 (C_q_, Cl–C_6_H_4_–CO–Ind–CH_2_–**CO**ON=C-_3_, C-1′); 168.34 (C_q_, Cl–C_6_H_4_–**CO**–Ind–CH_2_–COON=C-_3_, C-12′); 162.47 (C_q_, C-3); 156.07 (C_q_, Cl–C_6_H_4_–CO–**Ind**–CH_2_–COON=C-_3_, C-6′); 143.83 (C_q_, C-13); 139.42 (C_q_, Cl–**C_6_H_4_**–CO–Ind–CH_2_–COON=C-_3_, C-16′); 136.45 (C_q_, –COOCH_2_**C_6_H_5_**); 136.25 (C_q_, Cl–C_6_H_4_–CO–**Ind**–CH_2_–COON=C-_3_, C-4′); 135.25 (C_q_, Cl–C_6_H_4_–CO–**Ind**–CH_2_–COON=C-_3_, C-10′); 133.88 (C_q_, Cl–**C_6_H_4_**–CO–Ind–CH_2_–COON=C-_3_, C-13′); 132.15 × 2 (2 × CH, Cl–**C_6_H_4_**–CO–Ind–CH_2_–COON=C-_3_, C-14′ and C-18′); 130.93 (C_q_, Cl–C_6_H_4_–CO–**Ind**–CH_2_–COON=C-_3_, C-9′); 129.12 × 2 (2 × CH, Cl–**C_6_H_4_**–CO–Ind–CH_2_–COON=C-_3_, C-15′ and C-17′); 128.45 × 2 (2 × CH, –COOCH_2_**C_6_H_5_**); 127.98 × 2 (2 × CH, –COOCH_2_**C_6_H_5_**); 127.90 (CH, –COOCH_2_**C_6_H_5_**); 122.22 (CH, C-12); 115.01 (C_q_, Cl–C_6_H_4_–CO–**Ind**–CH_2_–COON=C-_3_, C-3′); 113.43 (CH, Cl–C_6_H_4_–CO–**Ind**–CH_2_–COON=C-_3_, C-8′); 111.77 (CH, Cl–C_6_H_4_–CO–**Ind**–CH_2_–COON=C-_3_, C-7′); 101.18 (CH, Cl–C_6_H_4_–CO–**Ind**–CH_2_–COON=C-_3_, C-5′); 65.91 (CH_2_, –COO**CH_2_**C_6_H_5_); 55.75 (CH_3_, –OCH_3_, C-19′); 46.75 (C_q_, C-17); 29.87 (CH_2_, Cl–C_6_H_4_–CO–Ind–**CH_2_**–COON=C-_3_, C-2′); 13.31 (CH_3_, C-11′); Ind = indole system. **^1^H NMR** (600 MHz, CDCl_3_) δ: 7.67 (2H, q_a_, *J* = 9.0 Hz, Cl–**C_6_H_4_**–CO–Ind–CH_2_–COON=C-_3_, C-_14′_–H and C-_18′_–H); 7.45 (2H, q_a_, *J* = 9.0 Hz, Cl–**C_6_H_4_**–CO–Ind–CH_2_–COON=C-_3_, C-_15′_–H and C-_17′_–H); 7.36–7.31 (5H, m, –COOCH_2_**C_6_H_5_**); 6.97 (1H, d, *J* = 2.4 Hz, Cl–C_6_H_4_–CO–**Ind**–CH_2_–COON=C-_3_, C-_8′_–H); 6.89 (1H, d, *J* = 9.0 Hz, Cl–C_6_H_4_–CO–**Ind**–CH_2_–COON=C-_3_, C-_5′_–H); 6.67 (1H, dd, *J* = 9.0, and 2.4 Hz, Cl–C_6_H_4_–CO–**Ind**–CH_2_–COON=C-_3_, C-_7′_–H); 5.31 (1H, t, *J* = 3.5 Hz, C_12_–H); 5.09 (2H, d, *J* = 12.6 Hz, –COO–C**H_2_**–C_6_H_5_); 3.83 (3H, s, –OCH_3_, C-_19′_–H_3_); 3.60 (2H, s, Cl–C_6_H_4_–CO–Ind–**CH_2_**–COON=C-_3_, C-_2′_–H_2_); 2.93 (1H, dd, *J* = 4.0, and 13.7 Hz, C-_18_–H_β_); 2.39 (3H, s, CH_3_, C-_11′_–H_3_); 1.16, 1.15, 0.94, 0.93, 0.92, 0.88, 0.88 (7 × 3H, 7 × s, 7 × CH_3_ groups); Ind = indole system. **MS/EI** (*m*/*z*): 15.02 (11%, CH_3_), 91.14 (7%, benzyl group), 112.01 (8%, C_6_H_4_Cl), 138.99 (100%, Cl–C_6_H_4_CO, main ion), 173.09 (5%, 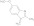), 357.79 (7%, indomethacin), 406.34 (39%, 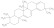), 790,41 (10%, 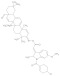), 898.61 (2%, M^•+^, molecular ion). **Elem. anal.**: for C_56_H_67_ClN_2_O_6_ calcd.: C: 74.77%, H: 7.51%, N: 3.11%, found: C: 74.73%, H: 7.50, %, N: 3.12%.

Conjugate of morpholide of oleanolic acid oxime and indomethacin, *3-indomethacinoxyiminoolean-12-en-28-oic acid morpholide* (**3d**):

**C_53_H_68_ClN_3_O_6_**. **Mol. mass**: 877.480. **Yield**: 815 mg (92.9 %). **M.p.**: 90–95 °C, amorphous powder. **IR** (KBr, cm^−1^) ν: 3330 (CH, Cl–**C_6_H_4_**–CO–**Ind**–CH_2_–COON=C-_3_), 1715 (C=O, Cl–C_6_H_4_–CO–Ind–CH_2_–**CO**ON=C-_3_); 1710 (N=C**,** Cl–C_6_H_4_–CO–Ind–CH_2_–COO**N=C**-_3_); 1630 (C=O, –**C(O)**Mor); 995 (C–N, –**C**(O)**Mor**); Mor = morpholine ring, Ind = indole system. **^13^C NMR** (151 MHz, CDCl_3_) δ: 175.46 (C_q_, Cl–C_6_H_4_–CO–Ind–CH_2_–**CO**ON=C-_3_, C-1′); 175.16 (C_q_, **–C(O)**Mor, C-28); 168.35 (C_q_, Cl–C_6_H_4_–**CO**–Ind–CH_2_–COON=C-_3_, C-12′); 162.40 (C_q_, C-3); 156.07 (C_q_, Cl–C_6_H_4_–CO–**Ind**–CH_2_–COON=C-_3_, C-6′); 144.66 (C_q_, C-13); 139.43 (C_q_, Cl–**C_6_H_4_**–CO–Ind–CH_2_–COON=C-_3_, C-16′); 136.28 (C_q_, Cl–C_6_H_4_–CO–**Ind**–CH_2_–COON=C-_3_, C-4′); 135.27 (C_q_, Cl–C_6_H_4_–CO–**Ind**–CH_2_–COON=C-_3_, C-10′); 133.87 (C_q_, Cl–**C_6_H_4_**–CO–Ind–CH_2_–COON=C-_3_, C-13′); 132.17 × 2 (2 × CH, Cl–**C_6_H_4_**–CO–Ind–CH_2_–COON=C-_3_, C-14′ and C-18′); 130.93 (C_q_, Cl–C_6_H_4_–CO–**Ind**–CH_2_–COON=C-_3_, C–9′); 129.08 × 2 (2 × CH, Cl–**C_6_H_4_**–CO–Ind–CH_2_–COON=C-_3_, C-15′ and C-17′); 121.49 (CH, C-12); 115.04 (C_q_, Cl–C_6_H_4_–CO–**Ind**–CH_2_–COON=C-_3_, C-3′); 113.41 (CH, Cl–C_6_H_4_–CO–**Ind**–CH_2_–COON=C-_3_, C-8′); 111.75 (CH, Cl–C_6_H_4_–CO–**Ind**–CH_2_–COON=C-_3_, C-7′); 101.12 (CH, Cl–C_6_H_4_–CO–**Ind**–CH_2_–COON=C-_3_, C-5′); 66.97 × 2 (CH_2_ × 2, –C(O)**Mor**); 55.75 (CH_3_, –OCH_3_, C-19′); 47.32 (C_q_, C-17); 46.03 (CH_2_, –C(O)**Mor**); 41.60 (CH_2_, –C(O)**Mor**); 29.91 (CH_2_, Cl–C_6_H_4_–CO–Ind–**CH_2_**–COON=C-_3_, C-2′); 13.29 (CH_3_, C-11′); Mor = morpholine ring, Ind = indole system. **^1^H NMR** (600 MHz, CDCl_3_) δ: 7.67 (2H, q_a_, *J* = 9.0 Hz, Cl–**C_6_H_4_**–CO–Ind–CH_2_–COON=C-_3_, C-_14′_–H and C_18′_-H); 7.45 (2H, q_a_, *J* = 9.0 Hz, Cl–**C_6_H_4_**–CO–Ind–CH_2_–COON=C-_3_, C-_15′_–H and C_17′_–H); 6.97 (1H, d, *J* = 2.4 Hz, Cl–C_6_H_4_–CO–**Ind**–CH_2_–COON=C-_3_, C-_8′_–H); 6.89 (1H, d, *J* = 9.0 Hz, Cl–C_6_H_4_–CO–**Ind**–CH_2_–COON=C-_3_, C-_5′_–H); 6.68 (1H, dd, *J* = 9. 0, and 2.4 Hz, Cl–C_6_H_4_–CO–**Ind**–CH_2_–COON=C-_3_, C-_7′_–H); 5.29 (1H, t, *J* = 3.5 Hz, C_12_-H); 3.85 (3H, s, –OCH_3_, C_19′_–H_3_); 3.69–3.53 (10H, m, –C(O)**Mor** + Cl–C_6_H_4_–CO–Ind–**CH_2_**–COON=C-_3_, C_2′_–H_2_); 3.09 (2H, d, *J* = 11.4 Hz, C_18_–H_β_); 2.37 (3H, s, CH_3_, C_11′_–H_3_); 1.16, 1.11, 1.06, 1.02, 0.92, 0.90, 0.76 (7 × 3H, 7 × s, 7 × CH_3_ groups); Mor = morpholine ring, Ind = indole system. **MS/EI** (*m*/*z*): 15.02 (14%, CH_3_), 85.06 (9%, morpholine), 91.14 (7%, benzyl group), 112.01 (10%, C_6_H_4_Cl), 138.99 (100%, Cl–C_6_H_4_CO, main ion), 173.09 (9%, 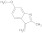), 357.79 (6%, indomethacin), 406.34 (36%, 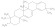), 790,41 (10%, 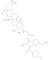), 878.59.61 (3%, M^•+^, molecular ion). **Elem. anal.**: for C_53_H_68_ClN_3_O_6_ calcd.: C: 72.45%, H: 7.80%, N: 4.78%, found: C: 72.43%, H: 7.82, %, N: 3.76%.

### 3.2. Biological Assays 

#### 3.2.1. Cell Culture and Viability Assay

HepG2 (ATCC HB 8065) and THLE-2 (ATCC CRL-2706) cells were provided by the American Type Culture Collection (ATCC, Gaithersburg, MD, USA). HepG2 cells were maintained in Dulbecco’s Modified Eagle’s Medium (DMEM, Sigma-Aldrich, St. Louis, MO, USA) containing 10% fetal bovine serum (EURx, Gdansk, Poland) and 1% antibiotic solution (Sigma-Aldrich, USA), while THLE-2 was cultured in BEGM supplemented with BulletKit (Lonza, Basel, Switzerland) and 10% fetal bovine serum, 5 ng/mL EGF, and 70 ng/mL phosphoethanolamine at 37 °C, in a humidified 5% CO_2_ atmosphere. To assess the effect of IND and OAO–IND derivatives on measured parameters, 1 × 10^6^ cells were seeded per 100 mm culture dish, and after 24 h of initial incubation, the cells were treated with 10 and 20 µM IND and its OAO conjugates, **3a**, **3b**, **3c**, and **3d**, and 0.1% dimethyl sulfoxide (DMSO) control solution. Incubation lasted for 24 h, and the cells were harvested. 

The effect of indomethacin and its tested derivatives on cell viability was assessed by MTT assay, following the standard protocol. Briefly, THLE-2 and HepG2 cells were seeded (1 × 10^4^ per well) in 96-well plates. After 24 h of pre-incubation in complete medium, compounds were added in various concentrations, and cells were incubated for 24 h. Later, the cells were washed twice with phosphate-buffered saline (PBS) and further incubated for 4 h with a medium containing 0.5 mg/mL 3-(4,5-dimethylthiazol-2-yl)-2,5-diphenyl-2H-tetrazolium bromide (MTT). Then, the formazan crystals were dissolved in acidic isopropanol, and the absorbance was measured at 570 and 690 nm. All experiments were repeated three times. In all following experiments, we used nontoxic concentrations of compounds (with viability level above 70%): 10 and 20 μM of **IND** and its OAO derivatives (**3a**, **3b**, **3c**, and **3d**).

#### 3.2.2. Nuclear and Cytosolic Fractions Preparation

The subcellular extracts from HepG2 and THLE-2 cells were prepared using the Nuclear/Cytosol Fractionation Kit (BioVision Research, MountainView, CA, USA) according to the manufacturer’s protocol. Protein concentration was assessed, and the samples were stored at −80 °C for further analysis.

#### 3.2.3. Total RNA Isolation and cDNA Synthesis 

The extraction of total RNA was performed by the GeneMatrix Universal DNA/RNA/Protein Purification Kit (EURx, Poland), and subsequently, samples were subjected to reverse transcription by the RevertAid First Strand cDNA Synthesis Kit (Thermo Fisher Scientific, Waltham, MA, USA), according to the manufacturer’s instructions.

#### 3.2.4. Quantitative Real-Time PCR

For the quantitative RT-PCR analyses, the Maxima SYBR Green Kit (Fermentas Inc., USA) and the Bio-Rad Chromo4 thermal cycler (Bio-Rad Laboratories, Hercules, CA, USA) were used. Protocol started with 5 min enzyme activation at 95 °C, followed by 40 cycles at 95 °C for 15 s, 56 °C for 20 s, and 72 °C for 40 s, and the final elongation at 72 °C for 5 min. Melting curve analysis was used for amplicon verification. The expressions of *TBP* (*TATA-box-binding protein*) and *PBGD* (*porphobilinogen deaminase*) were used to normalize the data. The Pfaffl comparative method was used for fold-change quantification. Primers were designed using the Beacon Designer software and subjected to a BLAST search to minimize unspecific binding. Only the primer pairs that generated intron-spanning amplicons were selected. The sequences of primers used to analyze *Nrf2*, *NF-κBp65*, *NF-κBp50*, *SOD-1*, *NQO1*, *COX-2*, *TBP*, and *PBGD* genes are listed in Table 2. 

#### 3.2.5. Western Blot Analysis

Cytosolic extracts for Nrf2, NF-κBp65, NF-κBp50, SOD-1, NQO1, COX-2, and β-actin, or nuclear extracts for Nrf2, NF-κBp65, NF-κBp50, and lamin protein detection, were separated on 12% or 10% SDS-PAGE slab gels, and proteins were transferred to the nitrocellulose Immobilon P membrane. After blocking for 2 h with 10% skimmed milk, proteins were probed with primary antibodies against Nrf2, NF-κBp65, NF-κBp50, SOD-1, NQO1, COX-2, β-actin, and lamin. β-actin and lamin served as loading controls. Alkaline phosphatase (AP)-labeled anti-rabbit IgG, anti-goat IgG, anti-mouse IgG secondary antibodies (Bio-Rad Laboratories, USA), and horseradish peroxidase (HRP)-conjugated anti-mouse IgG (Boster Bio, Pleasanton, CA, USA) secondary antibodies were used in the staining reaction. Bands were visualized by the AP Conjugate Substrate Kit NBT/BCIP or the chemiluminescent HRP substrate Clarity ECL Kits (Bio-Rad Laboratories, USA). The amount of immunoreactive products in each lane was determined using Quantity One software (Bio-Rad Laboratories, USA). Values were calculated as relative absorbance units (RQ) per mg of protein.

#### 3.2.6. Nrf2 and NF-κB Binding Assay

Nrf2, NF-κBp50, and NF-κBp65 activation were assessed by enzymatic immunoassay (Transcription Factor ELISA Assay Kit, Active Motif, Belgium) according to the manufacturer’s instructions. Activated Nrf2 was evaluated based on the amount of Nrf2 contained in the DNA-binding complex to the ARE sequence. The oligonucleotides containing the ARE consensus-binding site (5′-GTCACAGTGACTCAGCAGAATCTG-3′) for Nrf2 were immobilized on microplates as bait. Activated NF-κB was measured as the amount of p65 and p50 subunits held in the DNA-binding complex. The oligonucleotides containing (5’-GGGACTTTCC-3′) a consensus site for NF-κB were immobilized on microplates as bait. Nuclear fractions were incubated with oligonucleotides for 1 h. Then wells were washed, and DNA-bound subunits were detected by the specific primary antibody and a secondary antibody conjugated with the HRP. The results were expressed as the normalized level of absorbance (OD450 nm per mg of protein).

#### 3.2.7. Cell Cycle Distribution

The analysis of the cell cycle distribution was performed using the Muse^®^ Cell Cycle Kit (Merck, Germany) according to the manufacturer’s protocol. Briefly, cells (3 × 10^5^ per well) were grown in 6-well plates. After 24 h, the tested compounds were added, and the cells were incubated for a further 24 h. Topotecan (100 nM)-treated cells were used as a positive control of the cell cycle arrest. Subsequently, the cells were harvested by trypsinization, washed with PBS buffer, fixed in 70% ethanol, and frozen until staining at −20 °C. After overnight storage, the cells were washed with PBS buffer, stained with propidium iodide in the presence of RNase A, and the fluorescence signal was analyzed by flow cytometry on the Muse^®^ Cell Analyzer. Data were evaluated using Muse^®^ 1.5 Analysis software.

#### 3.2.8. Apoptosis

The externalization of phosphatidylserine was applied as a marker of apoptosis and analyzed by annexin V staining using the Muse^®^ Annexin V and Dead Cell Kit (Merck, Germany) according to the manufacturer’s protocol. The 7-aminoactinomycin D (7-AAD) stain was applied as a counterstain to discriminate between early and late apoptotic cells. The cells were grown for 24 h in the presence of the tested compounds. Topotecan (1500 nM)-treated cells were used as a positive control. Subsequently, the cells were collected and stained with annexin V and 7-AAD solution. After 20 min of incubation, the cells were analyzed by flow cytometry on the Muse^®^ Cell Analyzer, and data were evaluated using Muse^®^ 1.5 Analysis software.

#### 3.2.9. Proliferation

The Ki67 protein was used as a marker of proliferating cells, and its expression was analyzed using the Muse^®^ Ki67 Proliferation Kit (Merck, Germany) according to the manufacturer’s protocol. Briefly, cells (3 × 10^5^ per well) were seeded in 6-well plates. After 24 h, the tested compounds were added, and the cells were incubated for a further 24 h. Cells cultured in a medium without FBS (starved cells) were used as a positive control. The Ki67 protein expression was applied for the assessment of the cell proliferation rate. After the incubation, the cells were collected by trypsinization, washed with PBS buffer, instantly fixed, and exposed to the permeabilization buffer. After incubation with the Muse^®^ Hu Ki67 Antibody, the cells were analyzed by flow cytometry on the Muse^®^ Cell Analyzer, and data were evaluated using Muse^®^ 1.5 Analysis software. 

#### 3.2.10. Statistical Analysis

Statistical analysis was performed using the GraphPad InStat program (GraphPad Software, USA), assuming the significance level of changes to be *p* < 0.05. Student’s *t*-test assessed the statistical significance between the experimental groups and the control.

## 4. Conclusion

The results of this study provided evidence that conjugation of IND with OAO derivatives enhanced its potential as a modulator of Nrf2-ARE and NF-κB, the major signaling pathways involved in the induction of cytoprotective proteins and inflammation, respectively. Among the OAO derivatives, compound **3d** with morpholide substituent at the C-17 position of the OAO molecule was the most efficient enhancer of IND activity. This compound conjugated with ASA was shown earlier as a potent anti-inflammatory agent in the animal model and inhibitor of NF-κB in HepG2 cells. Thus, the results of this study further confirmed its potential as anti-inflammatory therapeutic alone or in conjugation with NSAID such as IND in the prevention or therapy of liver cancer. 

Importantly, OAO–IND conjugates enhanced the Nrf2 activation and expression in normal immortalized hepatocytes but reduced it in HCC HepG2 cells. The induction of the Nrf2-ARE pathway leading to increased expression of antioxidant and detoxifying enzymes and proteins is considered to be one of the most important cancer chemoprevention strategies.

In contrast, sustained Nrf2 activation has been observed in HCC and was thought to facilitate its progression and aggressiveness [26]. Therefore, the reduced activation and expression of Nrf2, along with the reduced activation of NF-κB and COX-2 as a result of treatment with OAO–IND conjugates in HCC cells, may inhibit their growth and invasiveness. Such possibility is supported by their ability, particularly conjugate **3d**, to inhibit cell proliferation and induce apoptosis more efficiently than indomethacin, as demonstrated in this study. Moreover, reduced activation of Nrf2 in HCC cells may reverse chemo- and/or radioresistance often occurring in this type of cancer. In contrast, in the immortalized hepatocytes, the THLE-2 line, which reflected the early stages of carcinogenesis, the enhanced activation of Nrf2 and reduced NF-κB may inhibit this process.

Since OAO–ASP conjugates exerted an opposite effect (i.e., activation of the Nrf2-ARE pathway in HepG2 cells), the results of this study indicate that the type and chemical structure of NSAID may be critical for the activity of their hybrids with OAO derivatives.

## Figures and Tables

**Table 1:** Comparison of OAO–IND derivatives’ cytotoxicity in non-tumorigenic (THLE-2) and hepatoma cells (HepG2) *.

Compound	THLE-2	HepG2
IND	>150	>150
3a	79.5 ± 2.25	33.5 ± 4.61
3b	44 ± 1.22	34 ± 2.5
3c	41.5 ± 3.5	40 ± 5.03
3d	42.5 ± 3.6	38.5 ± 5.48

* The IC_50_ values (*n* = 3) ± SEM (µM) were calculated based on the dose-response curves assessed by the MTT assay.

**Table 2:** Primers used in the RT-PCR.

Gene	Forward Primer	Reverse Primer
*NRF2*	5′ATTGCTACTAATCAGGCTCAG	5′GTTTGGCTTCTGGACTTGG
*SOD-1*	5′CGACAGAAGGAAAGTAATG	5′TGGATAGAGGATTAAAGTGAGG
*NQO1*	5′CAATTCAGAGTGGCATTC	5′GAAGTTTAGGTCAAAGAGG
*NF-κB p50*	5′ATCATCCACCTTCATTCTCAA	5′AATCCTCCACCACATCTTCC
*NF-κB p65*	5′CGCCTGTCCTTTCTCATC	5′ACCTCAATGTCCTCTTTCTG
*COX-2*	5′CCTGTGCCTGATGATTGC	5′CAGCCCGTTGGTGAAAGC
*PBGD*	5′TCAGATAGCATACAAGAGACC	5′TGGAATGTTACGAGCAGTG
*TBP*	5′GGCACCACTCCACTGTATC	5′GGGATTATATTCGGCGTTTCG

## Data Availability

The data is contained within this article and Supplementary Material.

## Supplementary Materials

The following are available online at https://www.mdpi.com/1424-8247/14/1/32/s1, Figure S1: ^1^H NMR for compound **3a**, Figure S2: ^13^C NMR for compound **3a**, Figure S3: ^1^H NMR for compound **3b**, Figure S4: ^13^C NMR for compound **3b**, Figure S5: ^1^H NMR for compound **3c**, Figure S6: ^13^C NMR for compound **3c**, Figure S7: ^1^H NMR for compound **3d**, Figure S8: ^13^C NMR for compound **3d**.
