# Supplementary material for: The Effect of Novel Oleanolic Acid Oximes Conjugated with Indomethacin on the Nrf2-ARE And NF-κB Signaling Pathways in Normal Hepatocytes and Human Hepatocellular Cancer Cells
Source: Pharmaceuticals (Basel). 2020 Dec 31;14(1):32. doi: 10.3390/ph14010032 (PMC7824409; doi:10.3390/ph14010032)

Supplementary Materials

The Effect of Novel Oleanolic Acid Oximes Conjugated with Indomethacin on the Nrf2-ARE And NF-κB Signaling Pathways in Normal Hepatocytes and Human Hepatocellular Cancer Cells

Maria Narożna[1], Violetta Krajka-Kuźniak[1], Barbara Bednarczyk-Cwynar[2], Robert Kleszcz[1], Jacek Kujawski[2], Wanda Baer-Dubowska[1]*

[1] Department of Pharmaceutical Biochemistry, Poznan University of Medical Sciences, 4, Święcicki Street, 60-781 Poznań, Poland
[2] Department of Organic Chemistry, Poznan University of Medical Sciences, 6, Grunwaldzka Street, 60-780 Poznań, Poland

*Corresponding author
e-mail: baerw@ump.edu.pl, phone: (+48) 61 854 66 25, fax: (+48) 61 854 66 20

Content:

^{1}H NMR and **^{13}C NMR** spectra of new compounds: **3a**, **3b**, **3c** and **3d**

Figure S1. **^{1}H NMR** spectra of conjugate **3a**, *3-indomethacinoxyiminoolean-12-en-28-oic acid*

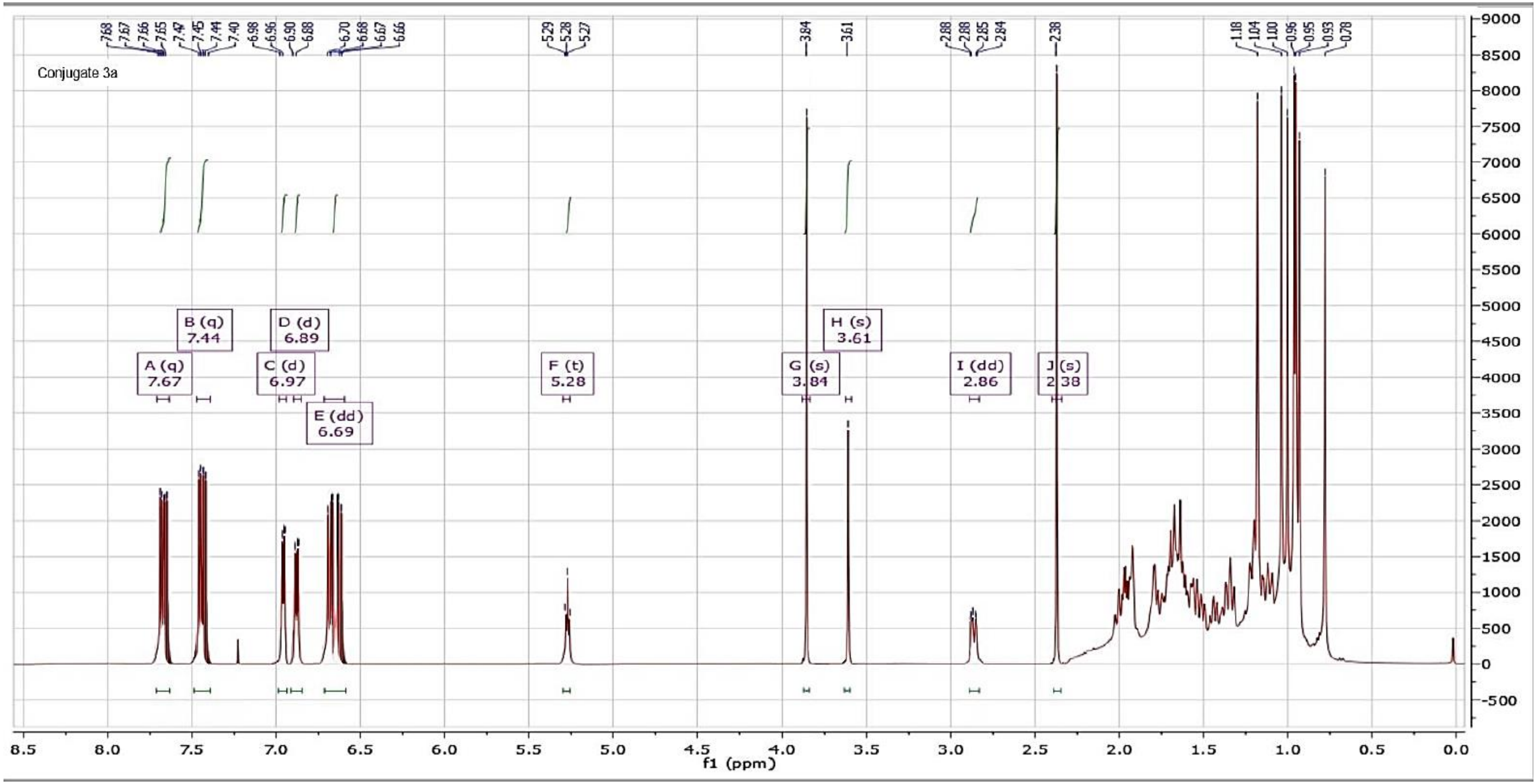

Figure S2. ^{13}**C NMR** spectra of conjugate **3a**, *3-indomethacinoxyiminoolean-12-en-28-oic acid*

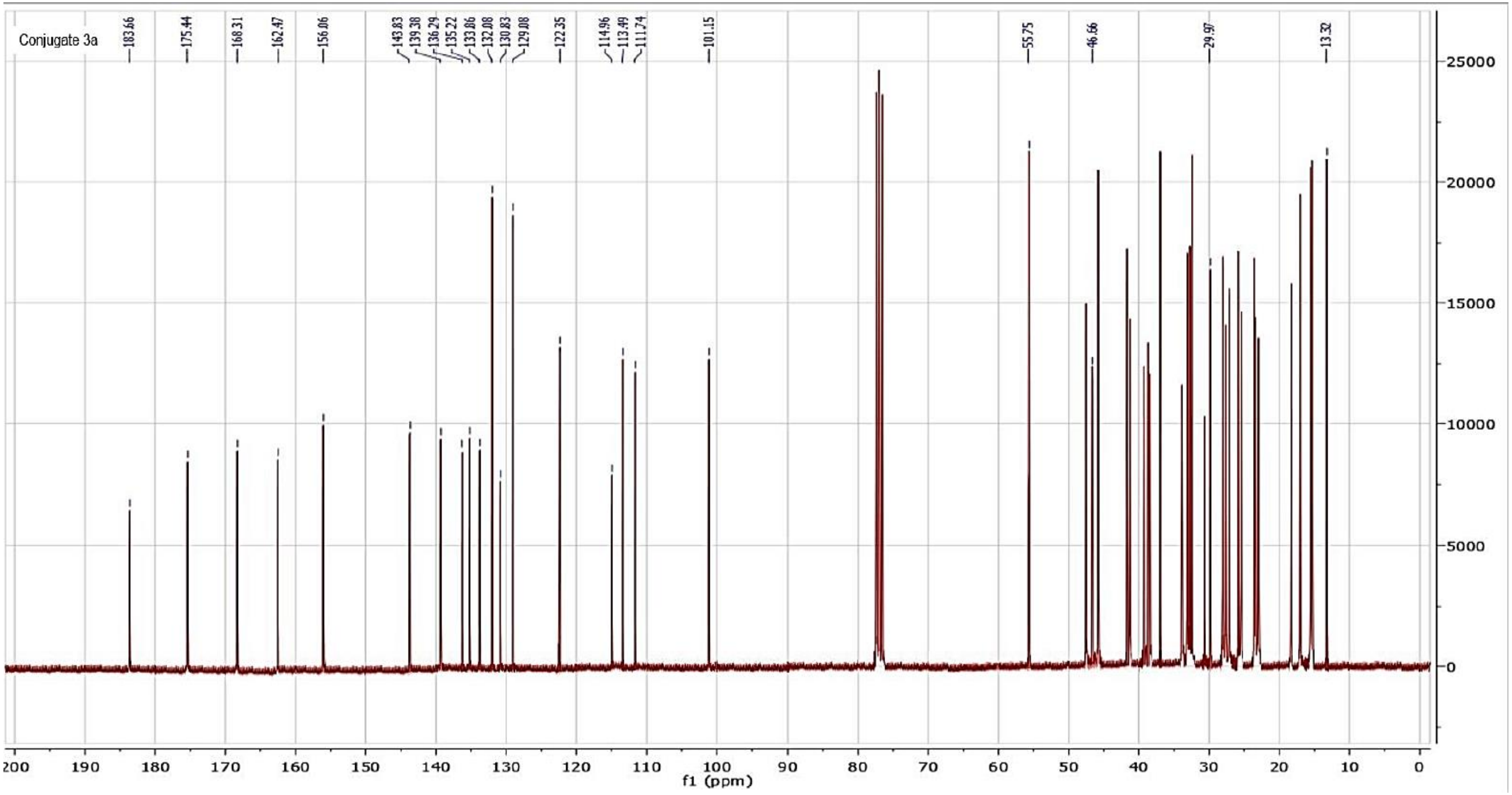

Figure S3. ^{1}**H NMR** spectra of conjugate **3b,** *3-indomethacinoxyiminoolean-12-en-28-oic acid methyl ester*

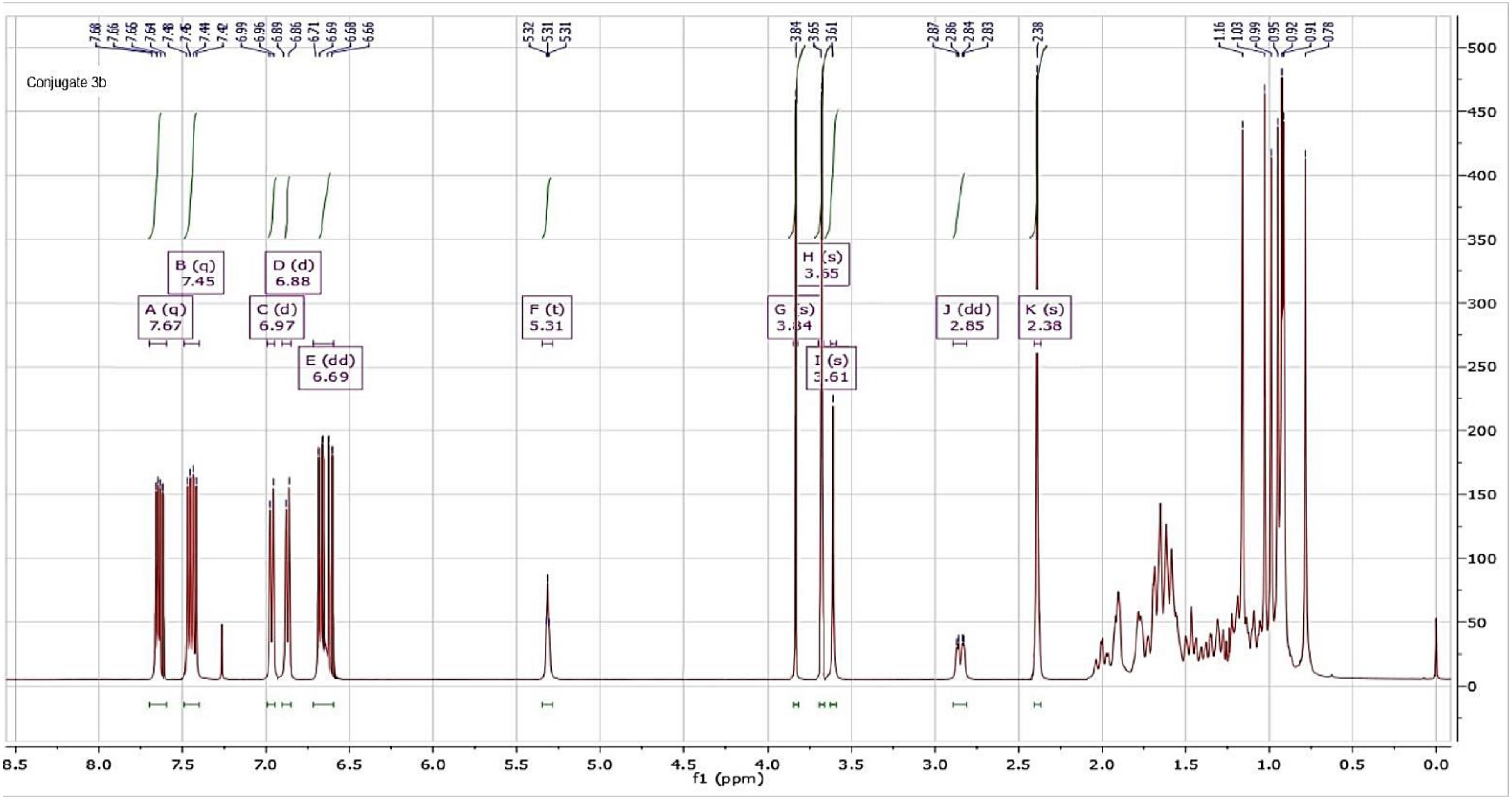

Figure S4. ^{13}**C NMR** spectra of conjugate **3b,** *3-indomethacinoxyiminoolean-12-en-28-oic acid methyl ester*

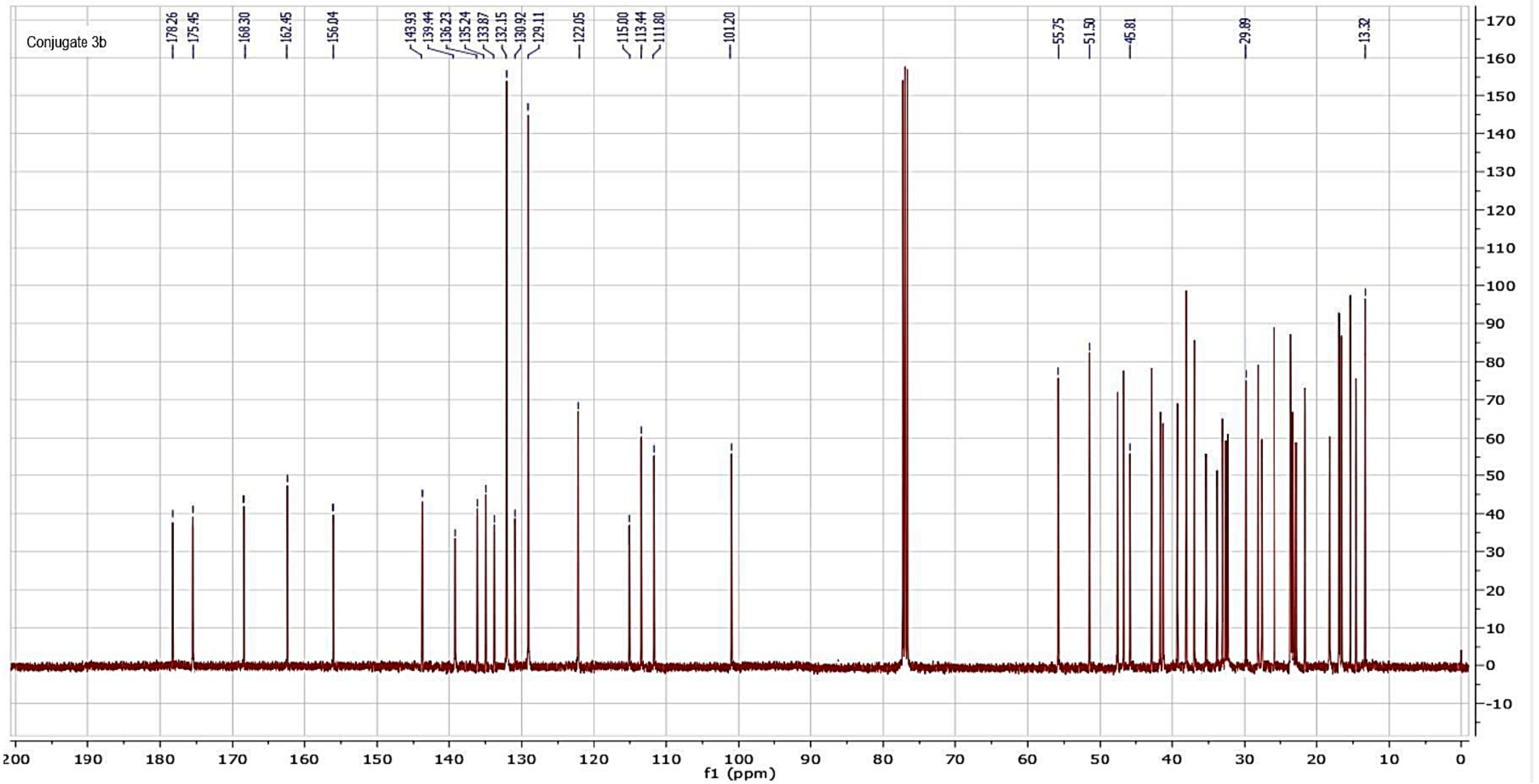

Figure S5. ^{1}H NMR spectra of conjugate **3c,** *3-indomethacinoxyiminoolean-12-en-28-oic acid benzyl ester*

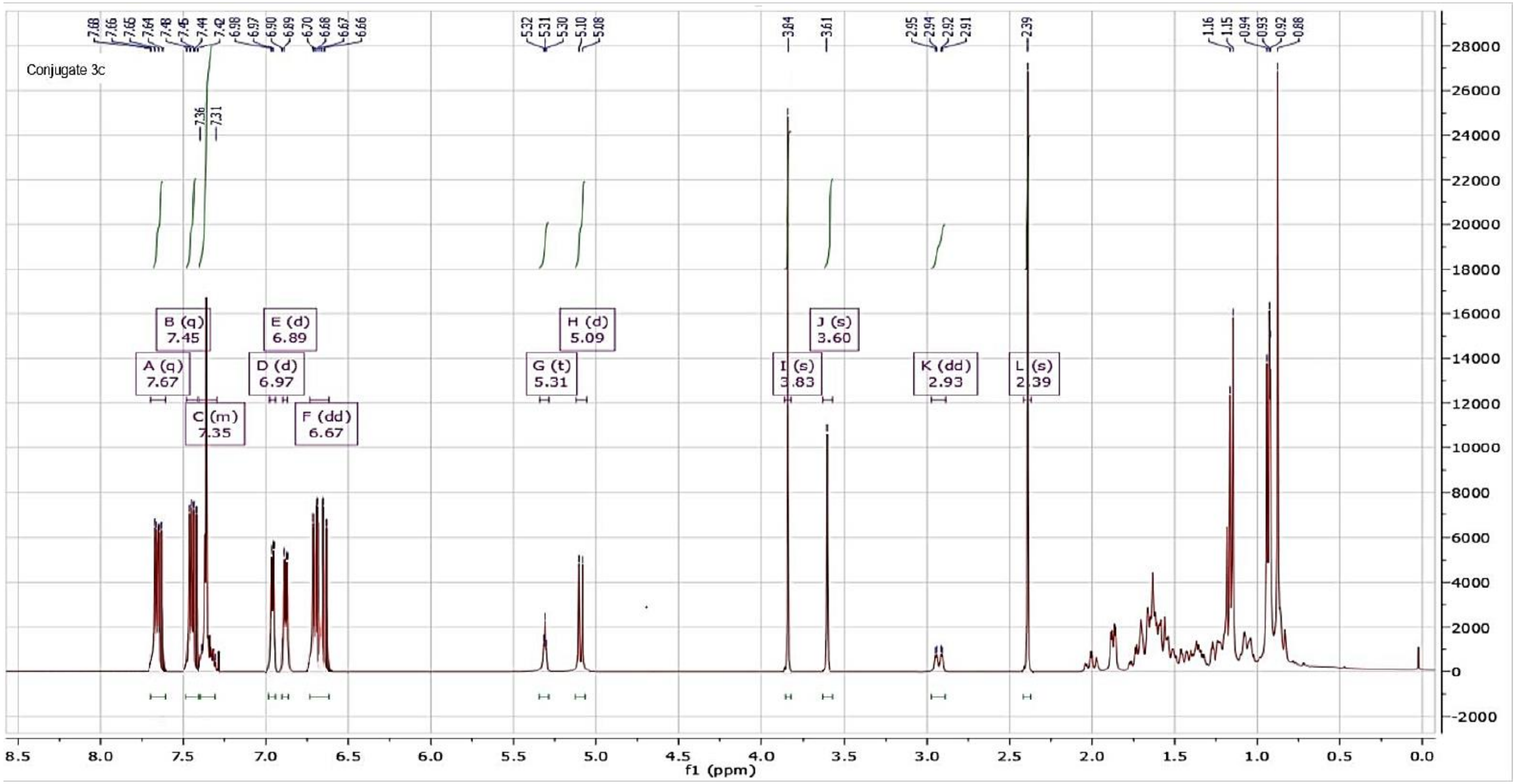

Figure S6. ^{13}C NMR spectra of conjugate **3c,** *3-indomethacinoxyiminoolean-12-en-28-oic acid benzyl ester*

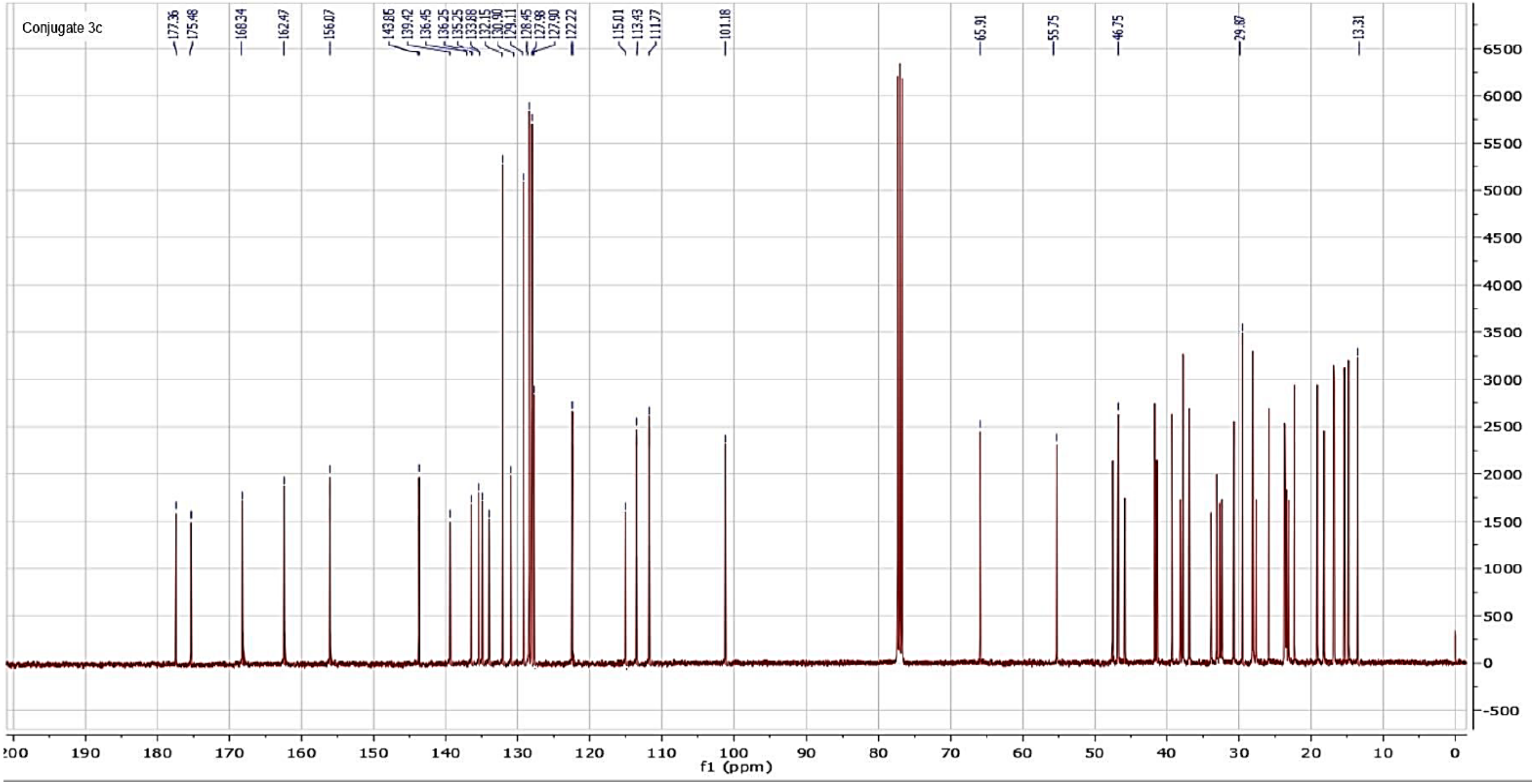

Figure S7. ^{1}**H NMR** spectra of conjugate **3d,** *3-indomethacinoxyiminoolean-12-en-28-oic acid morpholide*

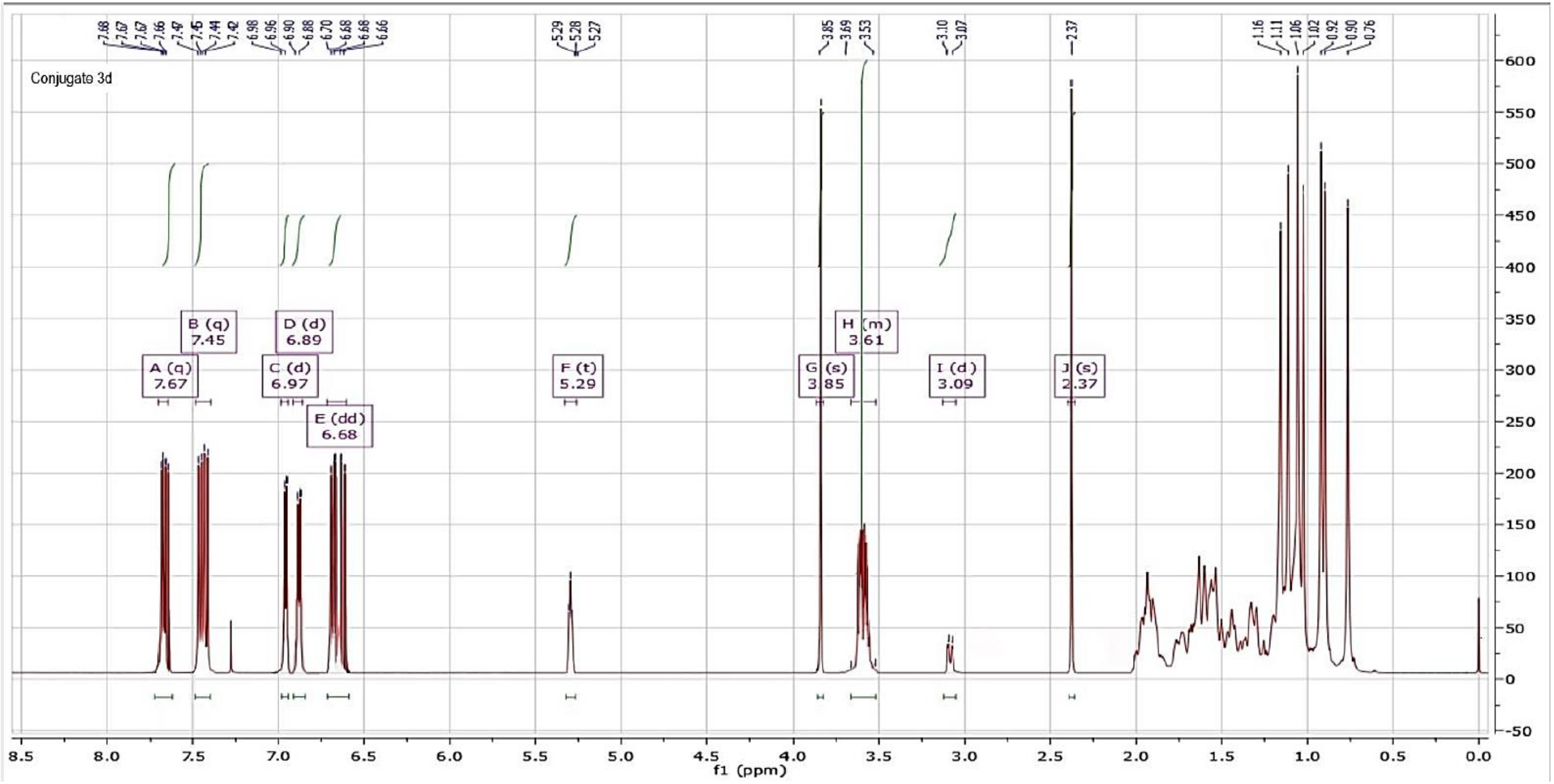

Figure S8. ^{13}**C NMR** spectra of conjugate **3d,** *3-indomethacinoxyiminoolean-12-en-28-oic acid morpholide*

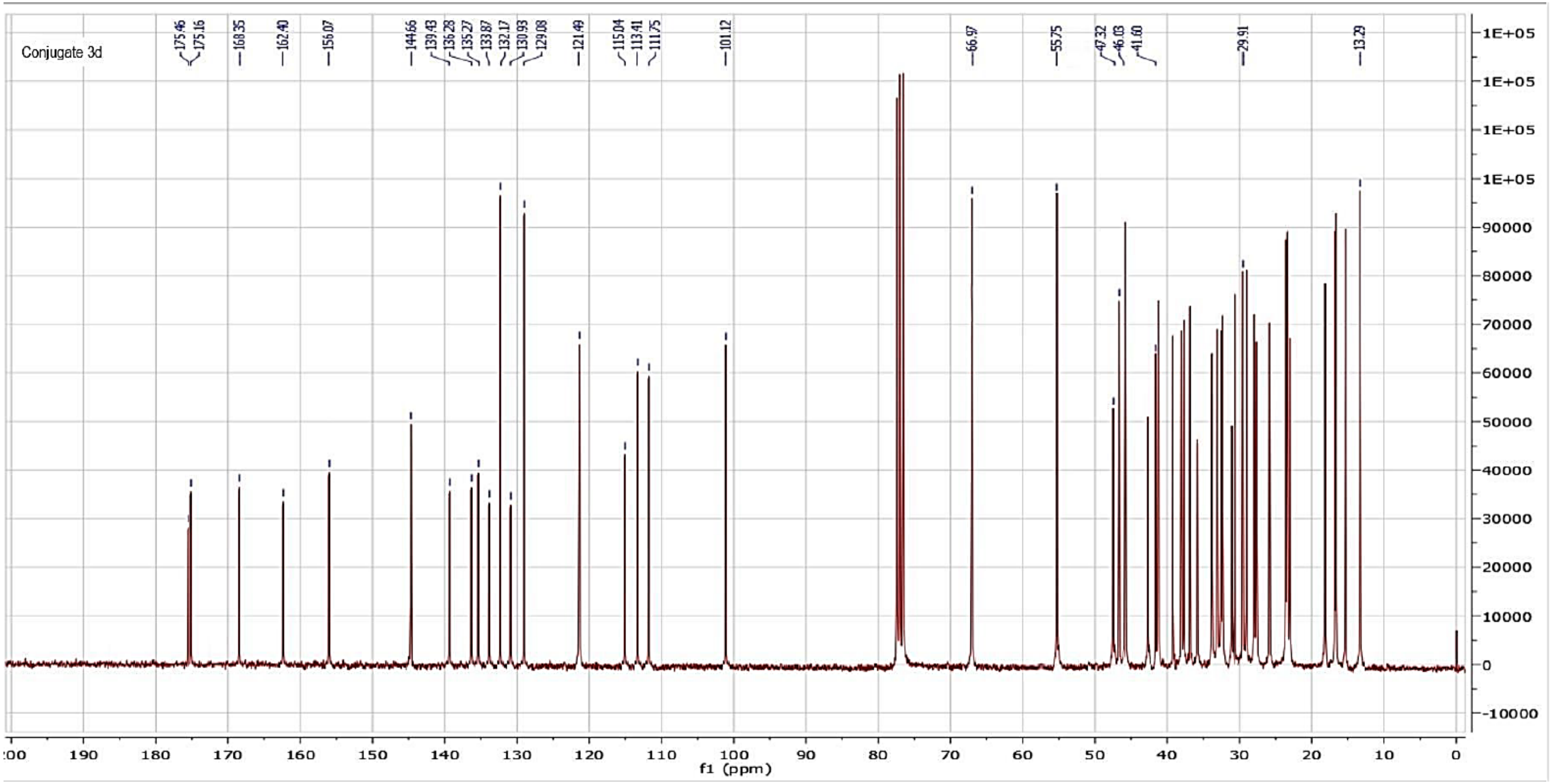

Supplement: Supplementary file 1 [file pharmaceuticals-14-00032-s001.pdf]
